# Exploring Nirmatrelvir Derivatives Through P2 Substituent Modifications and Warhead Innovations Targeting the Main Protease of SARS‐CoV‐2

**DOI:** 10.1002/ardp.70158

**Published:** 2025-11-29

**Authors:** Felipe Cardoso Prado Martins, Johannes Lang, Fernanda dos Reis Rocho, Xianxian Wang, Vinícius Bonatto, Jerônimo Lameira, Christian Klein, Carlos Alberto Montanari

**Affiliations:** ^1^ Medicinal and Biological Chemistry Group, São Carlos Institute of Chemistry University of São Paulo São Carlos São Paulo Brazil; ^2^ Medicinal Chemistry, Institute of Pharmacy and Molecular Biotechnology IPMB Heidelberg University Heidelberg Germany

**Keywords:** cysteine protease, Mpro inhibition, SARS‐CoV‐2, structure–activity relationships

## Abstract

The COVID‐19 pandemic underscored the urgent need for effective antiviral agents, particularly against coronaviruses, which pose a continuing threat of future outbreaks. Targeting the main protease (M^pro^), a key enzyme in viral replication, represents a promising therapeutic strategy. This study investigates structural modifications to known M^pro^ inhibitors, focusing on substitutions at the P2 position to explore alterations in both inhibitory potency and metabolic stability. Computational modeling and biochemical assays revealed that incorporating large, hydrophobic, and π‐rich groups, such as the 4‐phenylproline, significantly enhances binding affinity. Additionally, we evaluated warheads that have not yet been explored in the context of SARS‐CoV‐2 M^pro^ inhibition. Among these, fluoro‐vinylsulfone and nitrile groups demonstrated superior inhibitory activity. A fragment‐merging strategy combining an optimized P2 substituent with the nitrile warhead yielded a hybrid molecule with binding affinity comparable to nirmatrelvir. However, other analogs incorporating individual warhead optimizations displayed similar potency. These findings generate valuable insights into the design of robust M^pro^ inhibitors and support their potential development as broad‐spectrum antiviral agents.

## Graphical abstract

This study explores structural tuning of SARS‐CoV‐2 M^pro^ inhibitors through P2 position substitutions and novel warheads. Combining computational modeling and biochemical assays, we found that hydrophobic π‐rich groups and uncommon warheads like fluoro‐vinylsulfone or nitrile enhance potency. A fragment‐merged nitrile analog exhibited equipotent inhibitory activity to nirmatrelvir, offering new design insights for robust antiviral agents.

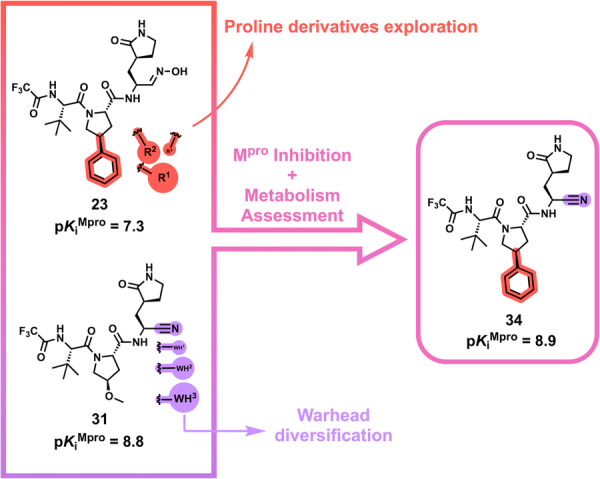

## 1 Introduction

Since its emergence in late 2019, COVID‐19 has rapidly proliferated across the globe. As of March 2025, 5 years after the WHO declared COVID as a pandemic, over 704 million cases have been reported worldwide, resulting in a staggering toll of over 7 million deaths [1]. This infectious disease was first identified in Wuhan, China, and subsequently attributed to the novel coronavirus, SARS‐CoV‐2, due to its genetic resemblance of approximately 79% to the earlier SARS‐CoV virus responsible for the 2002 SARS outbreak [2].

During the 2002 outbreak, rigorous public health measures, including patient isolation and contact tracing, were implemented to curb the spread of the virus [3]. Despite its genetic similarities to SARS‐CoV, the novel coronavirus is considerably more contagious, leading to its widespread and challenging containment in many nations [4]. Another coronavirus‐related illness, MERS, emerged in 2012, primarily afflicting populations in the Middle East and causing respiratory infections. In contrast to COVID‐19, MERS exhibited limited transmissibility, with only a few reported cases globally [5]. Additionally, various other coronaviruses, known to cause common colds, also pose risks of severe illness, particularly among children and the elderly [6, 7]. Consequently, ongoing research efforts are essential to develop effective treatments for these viral infections.

A well‐established strategy to tackle this issue is to develop inhibitors targeting enzymes that have vital importance to viral infection and replication within the host. This was already accomplished in the case of HIV, illustrating the potential for effective, safe, and successful treatments for viral diseases through targeted enzyme inhibition [8].

Pfizer developed Paxlovid®, the combination of nirmatrelvir and ritonavir, as a treatment for COVID‐19 [9]. Nirmatrelvir is an inhibitor of SARS‐CoV‐2 3CLpro, also called M^pro^, the main protease of the virus. Ritonavir acts as an inhibitor of the cytochrome P450 3A4 (CYP3A4) enzyme, which is part of the metabolism of xenobiotics in the human body [10, 11]. By inhibiting this enzyme, ritonavir slows down the metabolism of nirmatrelvir, leading to increased bioavailability.

The viral main protease plays a critical role in processing polyproteins, which are essential for viral replication and transcription. By inhibiting this crucial enzyme, the infectious process would ideally be terminated. Aside from its essential role, M^pro^ has no close homology in the human body, heightening its suitability as a target for inhibitor planning and synthesis by undermining the possibility of off‐target issues [12]. Mpro is a cysteine protease with a Cys‐His catalytic dyad [13].

Although the decline in COVID‐19 cases, the end of its pandemic status, and the availability of treatments and vaccines might suggest a reduced need for new drug candidates against SARS‐CoV‐2, several critical factors underscore the importance of continuing efforts to develop novel antiviral compounds.

It is crucial to acknowledge the possibility of mutations and, ultimately, resistance to antiviral medications, as it has already been observed in the case of vaccine‐resistant variants (Delta and Omicron) [14]. A study shows that the S1 region of M^pro^ is a likely region where mutations could occur, and such a subsite is detrimental to the recognition of inhibitors [15].

Furthermore, it is important to consider the possibility of a more infectious variant or even the emergence of a completely different virus, which could potentially lead to another pandemic, considering the rapid evolution of viruses [16].

Finally, another aspect worth considering is that Paxlovid comprises nirmatrelvir and ritonavir. Ideally, a novel M^pro^ drug candidate would exhibit a superior pharmacokinetic profile, avoiding the need for concurrent administration of a CYP inhibitor like ritonavir. A poor pharmacokinetic profile is one of the main reasons why drug candidates fail [17]. Addressing this key issue early on in drug discovery is fundamental to diminish attrition rates. Hence, optimization of the metabolic rate of a substance is a challenging endeavor that ought to be surpassed.

This was the main motivation for the study presented in this study where we developed tripeptidyl compounds based on the structures of one of our in‐house prototypes and nirmatrelvir, aiming at investigating the pharmacodynamics and pharmacokinetics toward M^pro^ inhibition. Apart from exploring the S2 subsite by harboring diverse proline analogs, we investigated different warheads that were previously applied in sorted cysteine protease inhibitors, but not yet in the context of SARS‐CoV‐2 M^pro^ inhibitors. These include oxime, the vinyl‐ and fluorovinylsulfones, and the newly developed *O*‐benzoyl oxime.

## 2 Results and Discussion

### 2.1 Design of New Inhibitors and Molecular Modeling

**Figure 1:**
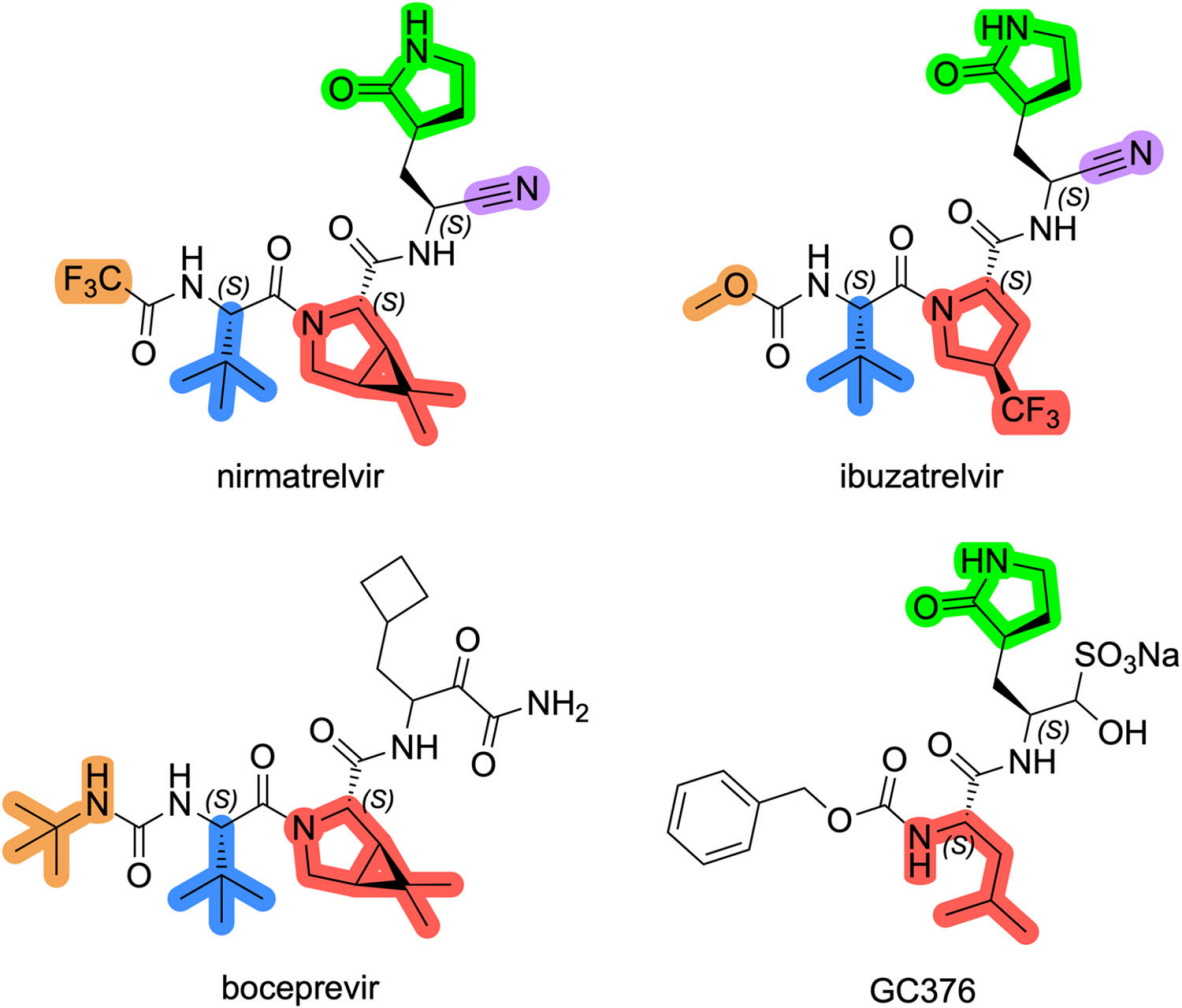
Chemical structures of previously explored inhibitors nirmatrelvir, ibuzatrelvir, boceprevir, and GC376.

Alongside nirmatrelvir in Figure 1 are the chemical structures of ibuzatrelvir, boceprevir, and GC376. These three compounds mimic the endogenous peptide substrate of M^pro^, with a characteristic Leu‐Gln‐Ser (Ala, Gly) preference for P2, P1, and P1’, respectively. Boceprevir was first developed to treat hepatitis C virus (HCV) infections [18]. GC376 was studied to treat feline infectious peritonitis (FIP) [19]. Both compounds act as inhibitors of their corresponding viral protease in each case. Both compounds were tested in cell‐based assays for the inhibition of the SARS‐CoV‐2 M^pro^, resulting in the micromolar range inhibition (IC_50_ = 8.0 ± 1.5 μM and IC_50_ = 0.15 ± 0.03 μM for boceprevir and GC376, respectively) [20].

As can be seen in Figure 1, nirmatrelvir incorporates groups from both molecules: the γ‐lactam at P1 position, the bicyclic proline at P2, and the tert‐butyl leucine nonnatural amino acid in P3. The innovative groups in nirmatrelvir were the trifluoroacetamide in P4 and the nitrile group as a warhead. The insertion of the trifluoroacetamide group was revealed to increase the compound's permeability [21], while the nitrile functions as a warhead by reacting with the catalytic Cys‐145 residue, in a reversible covalent way, producing a thioimidate complex.

The hydrophobic P2 group plays a crucial role in inhibiting the SARS‐CoV‐2 M^pro^. The bicyclic group in nirmatrelvir is highly effective; however, studies by Pfizer have revealed that this moiety is susceptible to oxidative metabolism by CYP3A4 enzymes [9].

Therefore, the primary objective of this study is to design and evaluate alternative proline‐based P2 groups that, while maintaining or enhancing potency against M^pro^, could exhibit improved metabolic stability compared with nirmatrelvir. More recently, Pfizer took the same approach and created the second‐generation oral treatment, ibuzatrelvir (Figure 1). This innovation directly addresses the metabolic issue by replacing the extensively used bicyclic proline found in earlier protease inhibitors.

Initially, we conducted covalent molecular docking and molecular dynamics (MD) simulations to investigate two alternative P2 groups as parent compounds. These groups were designed to explore variations in electronic and structural characteristics by incorporating a phenyl ring and a methoxy group attached to the proline‐based P2 moiety. Importantly, the nitrile warhead was retained in both designs, as it is a well‐established choice for reversible covalent inhibition of M^pro^, although we decided to explore other options further.

**Figure 2:**
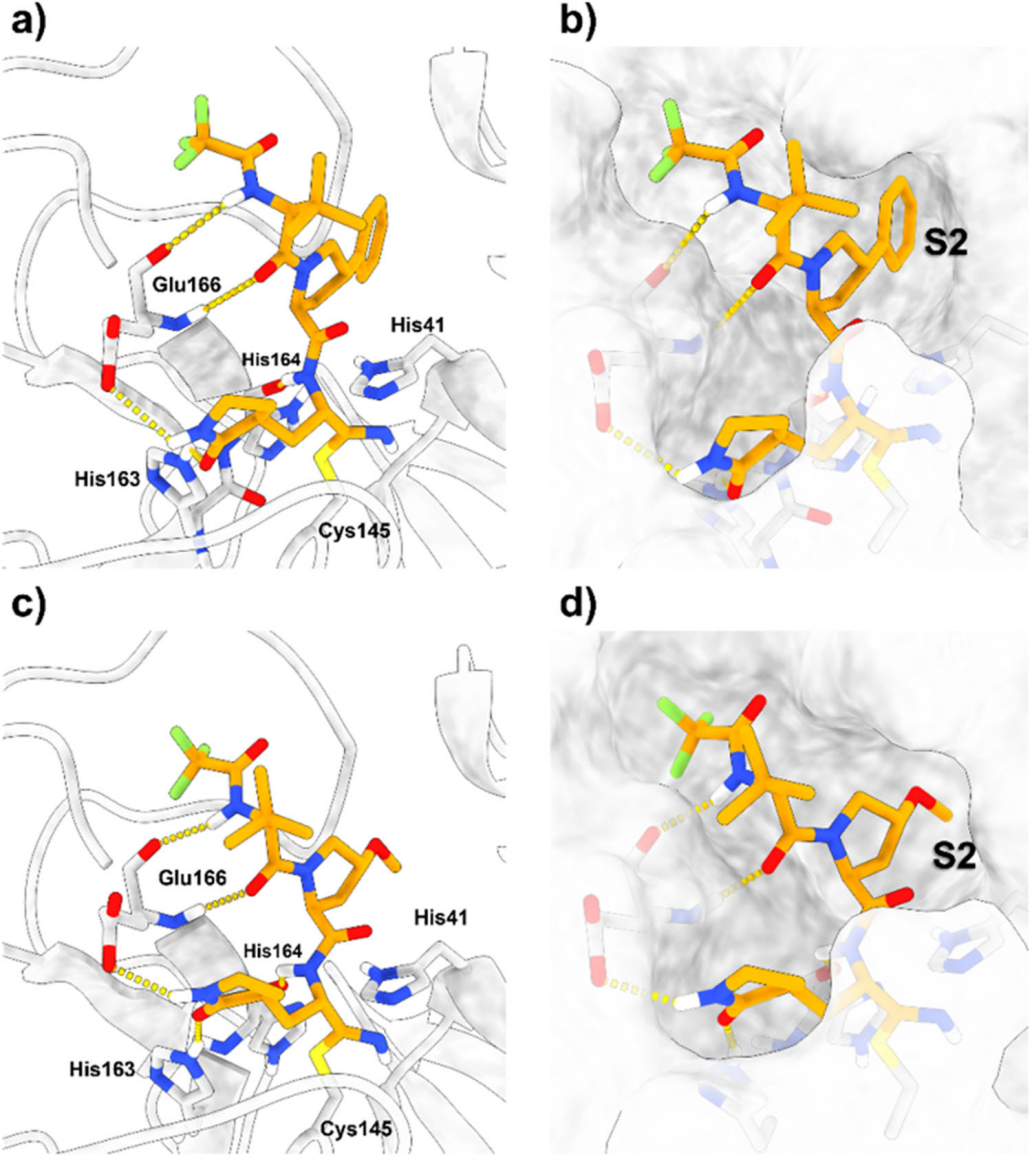
Covalent molecular docking of prototype compounds with different P2 moieties. (a) Putative binding pose of the compound containing a phenyl ring attached to the proline ring. Hydrogen bonds are depicted as yellow dashed lines. (b) Surface representation of M^pro^ interacting with the new phenyl‐proline compound. (c) Putative binding pose of the compound containing the methoxy group attached to the proline ring. Hydrogen bonds are depicted as yellow dashed lines. (d) Surface representation of M^pro^ interacting with the new methoxy‐proline compound. After 100 ns of MD simulations, both compounds demonstrated excellent stability within the M^pro^ active site (Figure 3). All expected hydrogen bonds with residues His163, His164, and Glu166 remained intact throughout the simulations. When the phenyl ring was attached to the proline at P2, this group penetrated deeper into the S2 cavity, allowing for π‐stacking interactions with the side chain of His41, as docking results suggested. Though the methoxy group also fit well into the S2 subpocket, its electronic properties prevented the formation of stacking interactions.

Covalent docking results showed that both compounds retained the same molecular interactions with M^pro^ as nirmatrelvir (Figure 2). Additionally, the phenyl and methoxy groups attached to the proline ring at P2 effectively occupy the S2 subpocket of M^pro^. Beyond the van der Waals interactions with nearby residues, the phenyl group was able to participate in π‐stacking interactions with the side chain of the catalytic His41.

**Figure 3:**
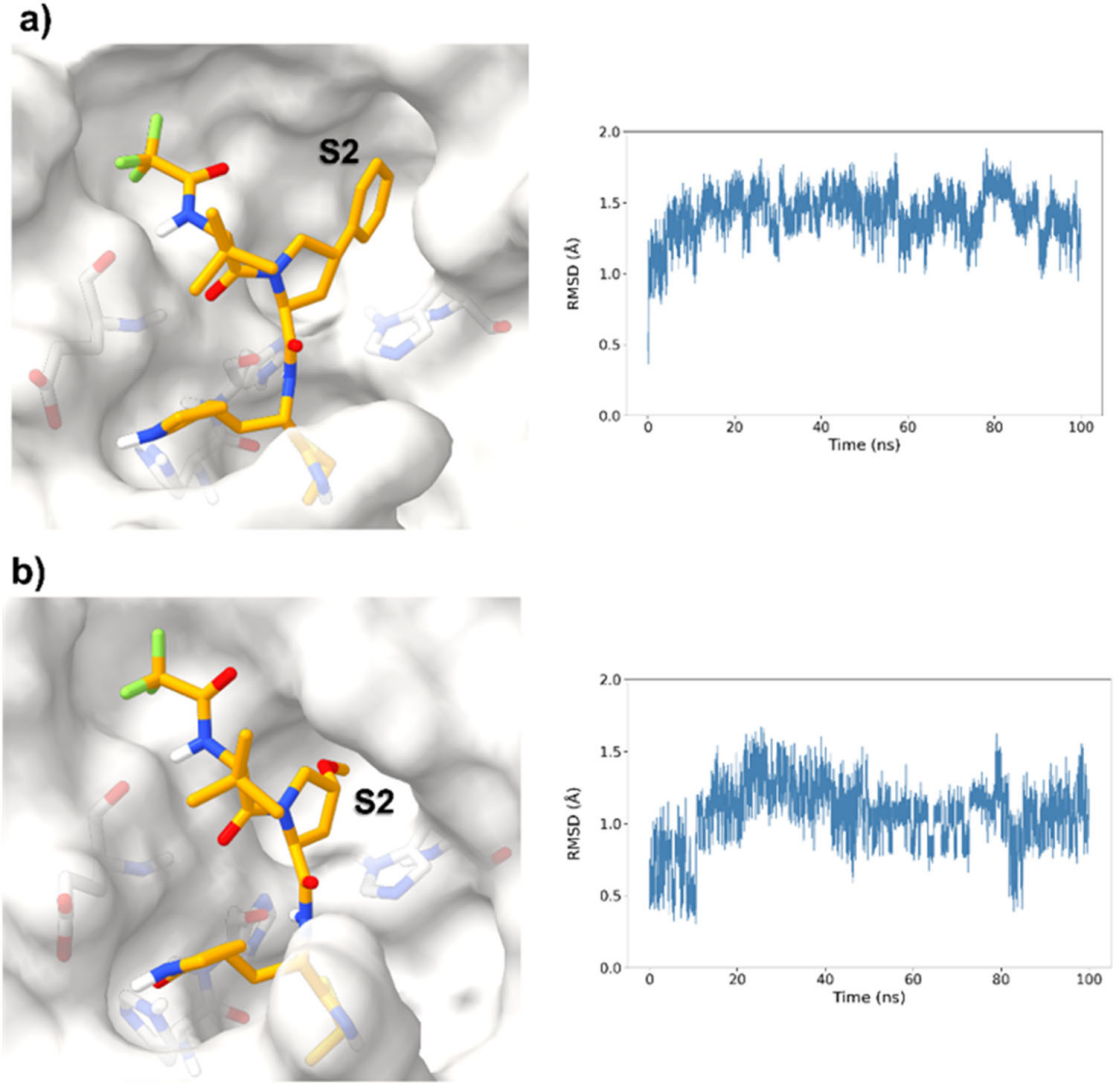
Surface representations from MD simulations after 100 ns of the proposed compounds with different P2 moieties, alongside the RMSD of the ligand over the trajectory. (a) M^pro^ interacting with the phenyl‐proline inhibitor. (b) M^pro^ interacting with the methoxy‐proline inhibitor.

After 100 ns of MD simulations, both compounds demonstrated excellent stability within the M^pro^ active site (Figure 3). All expected hydrogen bonds with residues His163, His164, and Glu166 remained intact throughout the simulations. When the phenyl ring was attached to the proline at P2, this group penetrated deeper into the S2 cavity, allowing for π‐stacking interactions with the side chain of His41, as docking results suggested. Though the methoxy group also fit well into the S2 subpocket, its electronic properties prevented the formation of stacking interactions.

**Figure 4:**
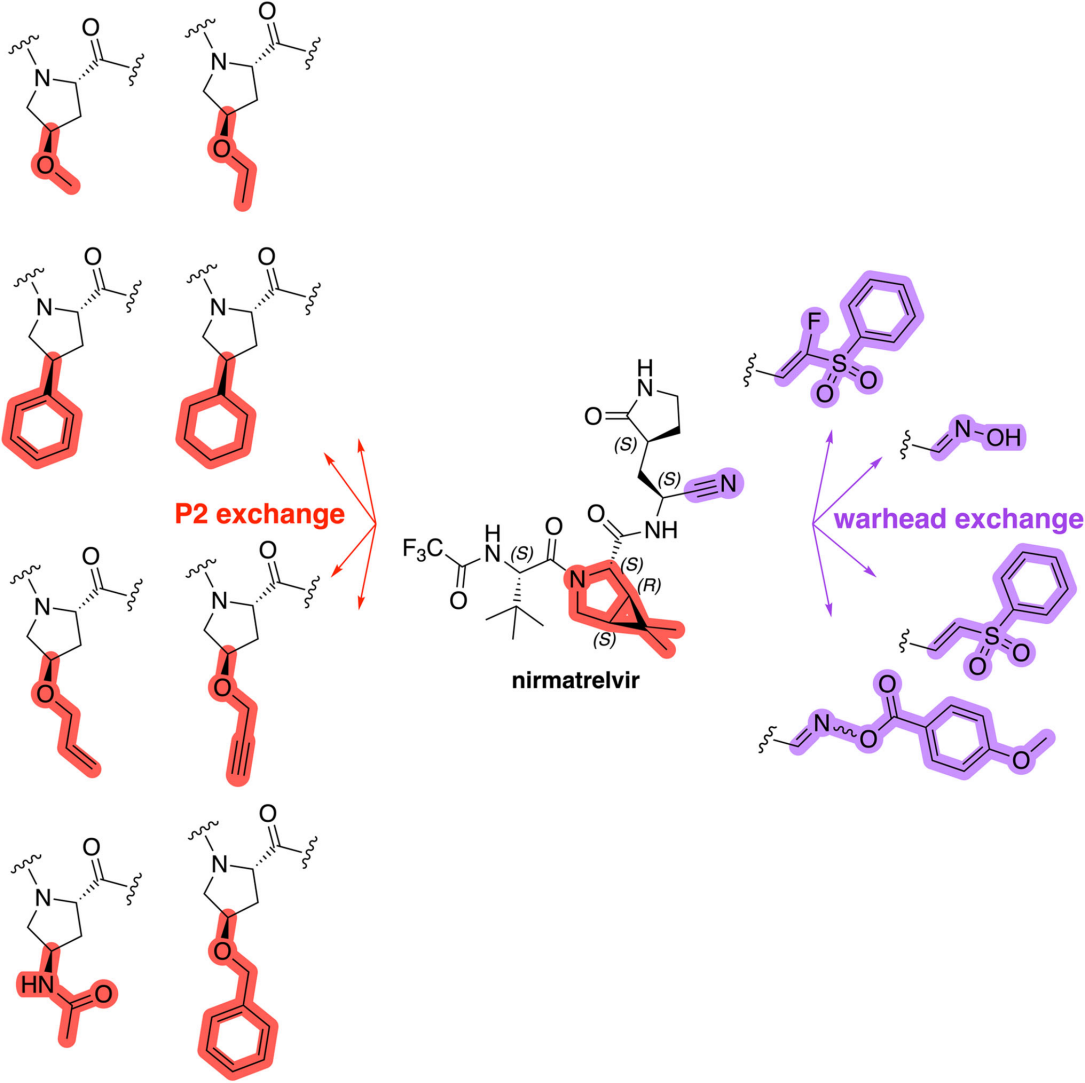
Exhibition of the chemical space surrounding the proline moiety and the identity of warheads employed to achieve the desired covalent bond formation.

The simulations suggested that the S2 subpocket can accommodate bulkier and lipophilic groups, such as the phenyl ring, and can likely tolerate π‐systems, allowing for stacking interactions. Based on these findings, we decided to explore new P2 groups (Figure 4) to test our hypothesis and to evaluate their metabolic stability. The proposed new groups aim to investigate the impact of electronic effects (e.g., replacing the phenyl ring with a cyclohexane ring). We also explored increasing the length of the methoxy group by adding additional carbon units, including unsaturated carbon atoms, to modify the electronic properties. We further introduced a hydrogen bond donor and acceptor by incorporating an amide bond in the group attached to the proline‐based P2. Finally, for the new compounds, we also decided to investigate new options beyond the well‐established nitrile warhead, including oxime, vinyl sulfone‐based groups, and the O‐benzoyl oxime. These electrophiles are commonly found in other cysteine protease inhibitors but were, to our knowledge, not yet employed in SARS‐CoV‐2 M^pro^ inhibition.

### 2.2 Chemistry

**Scheme 1:**
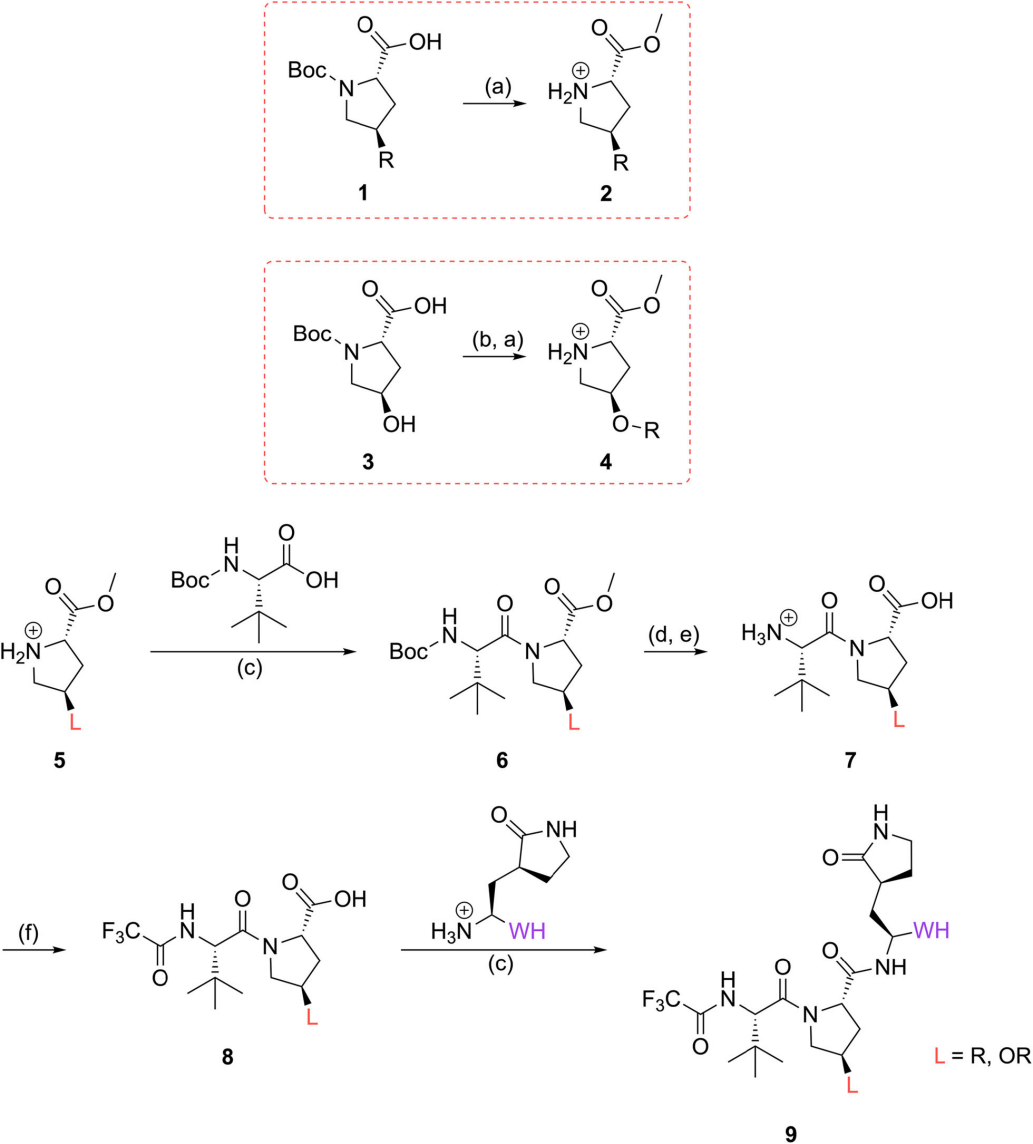
Synthesis of tripeptidyl compounds. Reagents and conditions: (a) SOCl_2_, MeOH, 0^o^C to r.t., 12 h; (b) primary alkyl halide, NaH, THF/DMSO (16:1), 0^o^C to r.t., 16 h; (c) HATU, DIPEA, DMF, r.t., 16 h; (d) LiOH, THF/H_2_O (1.7:1), r.t., 3 h; (e) TFA, DCM, 0 C to r.t., 30 min; (f) TFAA, DCM, r.t., 3 h.

The chemical synthesis of targeted compounds proceeded by classical peptide coupling reactions, as can be seen from Scheme 1.

Some commercially available Boc‐protected amino acids **1** were converted to their amino ester counterparts **2** by means of thionyl chloride (SOCl_2_) treatment (upper red dotted box) and used in the following step with no further purification. To increase the chemical diversity at the P2 position, Boc‐4‐hydroxyproline **3** was directly derivatized to alkoxy analogs employing primary alkyl halides and sodium hydride (NaH) [22] and subsequently transformed into the amino esters **4** as described above (lower red dotted box). To obtain the tripeptidyl scaffold, the appropriate proline amino ester **5** was coupled with Boc‐*tert*‐butyl leucine using HATU as coupling reagent to generate the synthetic intermediates **6**. The “L” group in Scheme 1 represents either substituents directly attached to the carbon cycle (R), that is, phenyl group, or groups attached to the oxygen atom from the hydroxyproline analogs (OR), that is, methoxy. These compounds had their ester groups removed by alkaline treatment with lithium hydroxide (LiOH) and Boc group removal promoted by trifluoroacetic acid (TFA), yielding compound **7**. The trifluoroacetamide group was introduced by the reaction with trifluoroacetic anhydride (TFAA) to obtain **8**, which was readily coupled with the appropriate lactam‐derivatized fragment to generate the desired tripeptidyl structure **9**.

**Scheme 2:**
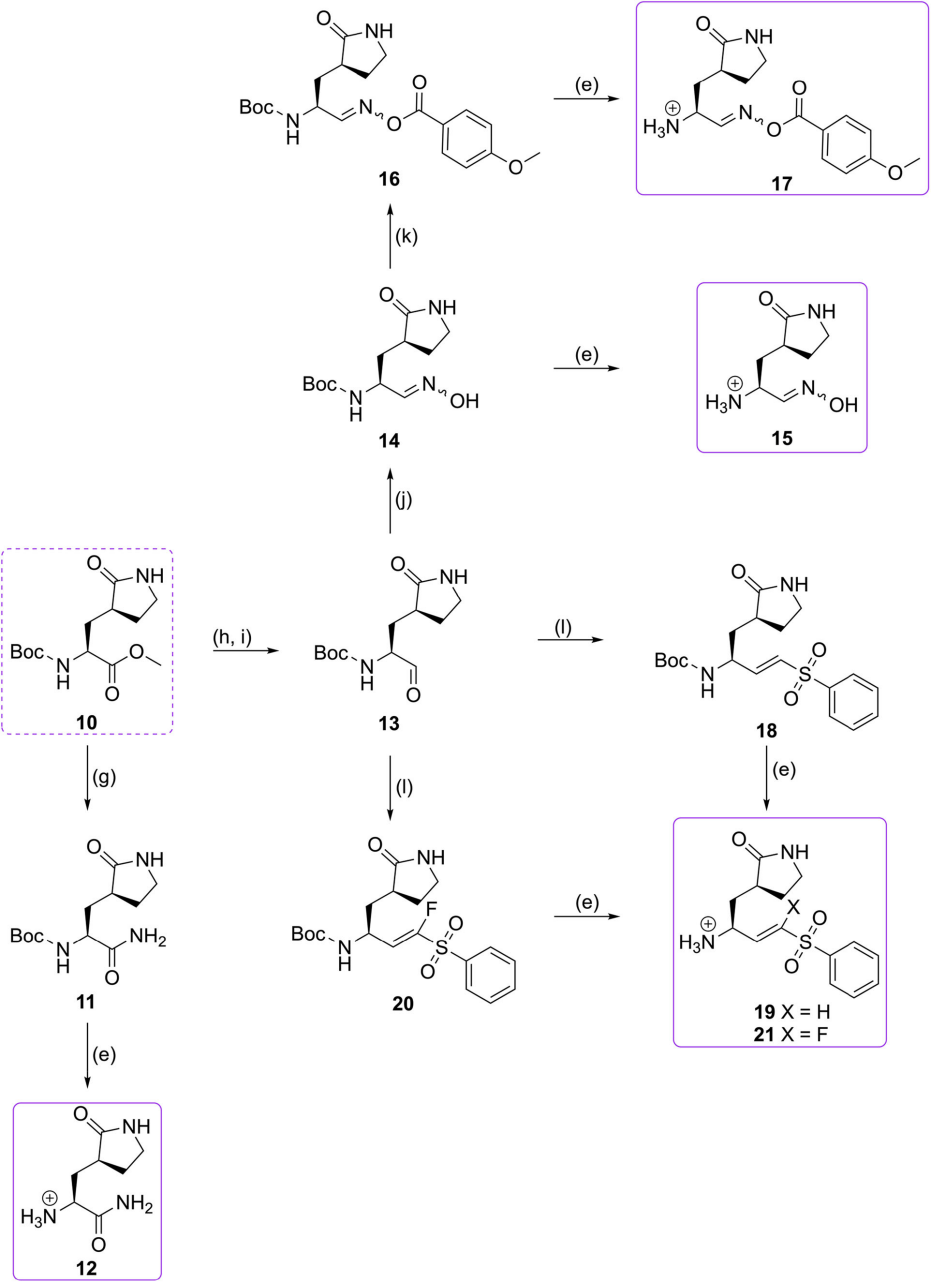
Introduction of varied warhead moieties. Reagents and conditions: (e) TFA, DCM, 0^o^C to r.t., 30 min; (g) 7 N NH_3_ in MeOH, r.t., 48 h; (h) NaBH_4_, MeOH, 0^o^C to r.t., 2.5 h; (i) DMP, DCM, r.t., 2.5 h; (j) NH_2_OH.HCl, NaHCO_3_, EtOH, r.t., 12 h; (k) appropriate carboxylic acid, DCC, DMAP, DCM, 0^o^C to r.t., 16 h; (l) appropriate sulfonyl phosphonate, LHMDS, THF, −78^o^C to r.t., 2 h.

To obtain the different warheads applied on this study (Scheme 2), we started from the commercially available γ‐lactam Boc‐amino acid ester **10**. To get the nitrile, the ester group of **10** was transformed into the amide **11** in one step using an ammonia in a methanol solution. The Boc amide was deprotected utilizing TFA to yield compound **12**, which was coupled with fragment **8**.

As for the remaining warheads, the ester lactam **10** was converted into its aldehyde form **13** by reduction to the alcohol with sodium borohydride, followed by oxidation with the Dess–Martin periodinane. From the aldehyde **13**, the oxime **14** was achieved by the reductive amination with hydroxylamine, while the acylated oxime **16** was obtained by the reaction with 4‐anisol carboxylic acid. The vinyl sulphone was obtained by combining **13** and the suitable sulfonyl phosphonate via a Horner–Wadsworth–Emmons olefination reaction. The same procedure was used to obtain the fluorovinylsulfone **20**, with the difference that the sulfonyl phosphonate was fluorinated using Selectfluor [23]. All final synthetic intermediates **15**, **17**, **19**, and **21** were attained by Boc deprotection with TFA.

### 2.3 Biochemical Evaluation and SAR Analysis

The generated compounds were tested and had their inhibitory activity compared with nirmatrelvir. All novel molecules produced can be considered direct molecular pairs to each other, as their only structural variation appears either on the derivatized proline ring at P2 or at the warhead group. This punctual approach facilitates the thorough observation of how each substituent anchored to the proline ring is capable of modulating affinity and chemical stability.

Nirmatrelvir was synthesized in‐house, and its p*K*_i_ was found to be > 8.0 [24]. It is important to note that a more precise determination of the *K*_i_ value is impractical due to the high affinity to the enzyme, which hampers the observation of a kinetic equilibrium. This goes in accordance with a previous publication [25].

Table 1 exhibits the results for the synthesized compounds. The primary synthesized compound was **22**, which harbors an *O*‐methoxy group attached to the proline ring and an oxime as warhead. All further modifications will be compared with this prototype compound and to nirmatrelvir. Substituents containing π‐electrons, such as **23**, **24**, and **25**, gave higher increases in p*K*_i_ values. The insertion of an O‐benzyl group, as in **29**, which has an aromatic moiety, resulted in an equipotent compound relative to **22**. A plausible explanation for this result would be the presence of a very bulky, lengthy, and rotatable group as compared with the other substituents mentioned above containing a π‐system. Such characteristics possibly hindered a proper fit and restricted the interaction with the amino acid residues located at S2. A second observation of this nature should be made to **28**, the amide‐linked proline derivative, which also possesses a conjugation pattern and an extra hydrogen bond donor group. However, it did not reveal the same magnitude increase in p*K*_i_ values as the previous compounds. As vastly explored, it is common for cysteine proteases and, in particular, human cathepsins, to have a detrimental preference for lipophilic groups at the S2 subsite, which is also the case for SARS‐CoV‐2 M^pro^. Hence, the introduction of a polar group in such a hydrophobic environment was not well tolerated.

**Table 1:** Compounds featuring the P2 substituent and warhead and each corresponding SARS‐CoV‐2 M^pro^ p*K*_i_ value.

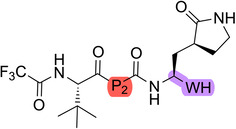			
Compound	P2	WH	p*K*_i_
**22**	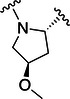	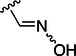	6.8 ± 0.01
**23**	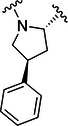	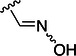	7.3 ± 0.01
**24**	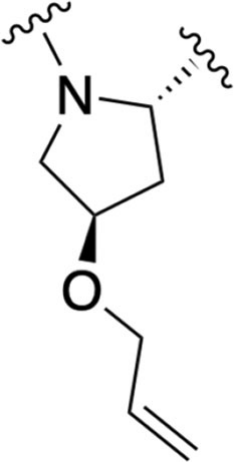	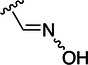	7.3 ± 0.08
**25**	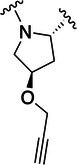	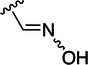	7.5 ± 0.04
**26**	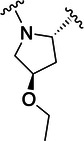	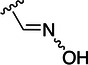	7.0 ± 0.02
**27**	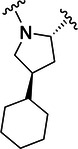	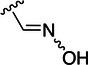	7.0 ± 0.02
**28**	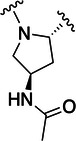	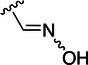	7.0 ± 0.03
**29**	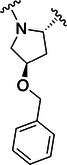	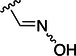	6.7 ± 0.02
**30**	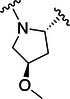	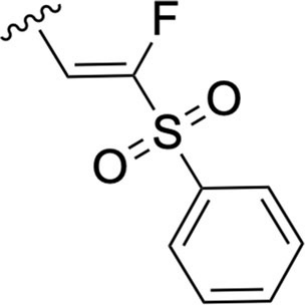	8.1 ± 0.01
**31**	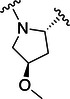	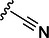	8.8 ± 0.01
**32**	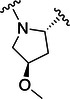	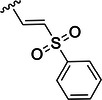	*K*_inact_/K_I_ = 4.4 × 10^3^ M^−1^ s^−1^ (±500 M^−1^ s^−^^1^)
**33**	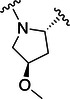	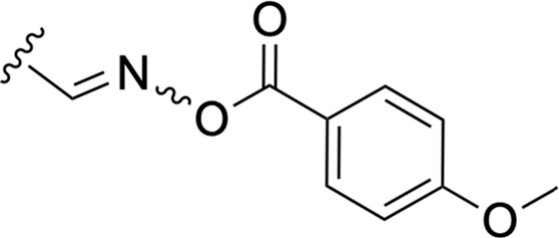	*K*_inact_/K_I_ = 7.7 × 10^4^ M^−1^ s^−1^ (±1930 M^−1^ s^−1^)

Increasing the chain length, that is, in **26**, or inserting a larger aliphatic cyclic ring, such as in **27**, were not relevant features to enhance the affinity of this structure.

The presence of a histidine moiety at S2 could potentially elucidate the increased affinity of M^pro^ toward compounds **23**, **24,** and **25** by creating either hydrophobic interactions or a perpendicular t‐shaped stacking. A similar effect is detected in the crystal structure of nirmatrelvir and M^pro^ for the bicyclic proline and in our initial computational results.

These results validate our initial hypothesis that proline derivatives containing lipophilic substituents are well tolerated when targeting M^pro^. Although increasing lipophilicity to magnify van der Waals interactions is a known strategy, one might have to consider the potential risk of a higher metabolic clearance by cytochrome P450 enzymes and the possibility of nonspecific hydrophobic interactions with deleterious targets.

The warhead exchange study, however, produced more appealing results: we surprisingly witnessed an activity cliff. Nitrile compound **31** is 100‐fold more potent (Δp*K*_i_ = 2.0) than its direct oxime analog **22**, revealing a distinct preference for this reactive group. This result, however, is backed up by the observations Keserű et al. made by performing the reaction of distinct warheads with glutathione (GSH). It was observed that only the nitrile suffered the nucleophilic attack from the sulfur atom in this tripeptide, while no reaction happened when an oxime was employed, indicating its lower reactivity [26]. This outcome contrasts with earlier results obtained by our group [27], when the oxime warhead generated a comparable potency relative to a nitrile warhead; additional studies would be required to confirm this trend.

The fluorinated version of the classical vinyl sulfones, as in **30**, exhibited a reversible behavior, as expected, being close to 20‐fold more potent (Δp*K*_i_ = 1.3) than its parent compound **22**. We also harbored the vinyl sulfone reactive group, creating **32**, an irreversible inhibitor.

Transforming the oxime to its acylated form as in **33** also produced an irreversible inhibitor, with close to 20‐fold higher reactivity than the vinyl sulfone.

**Figure 5:**
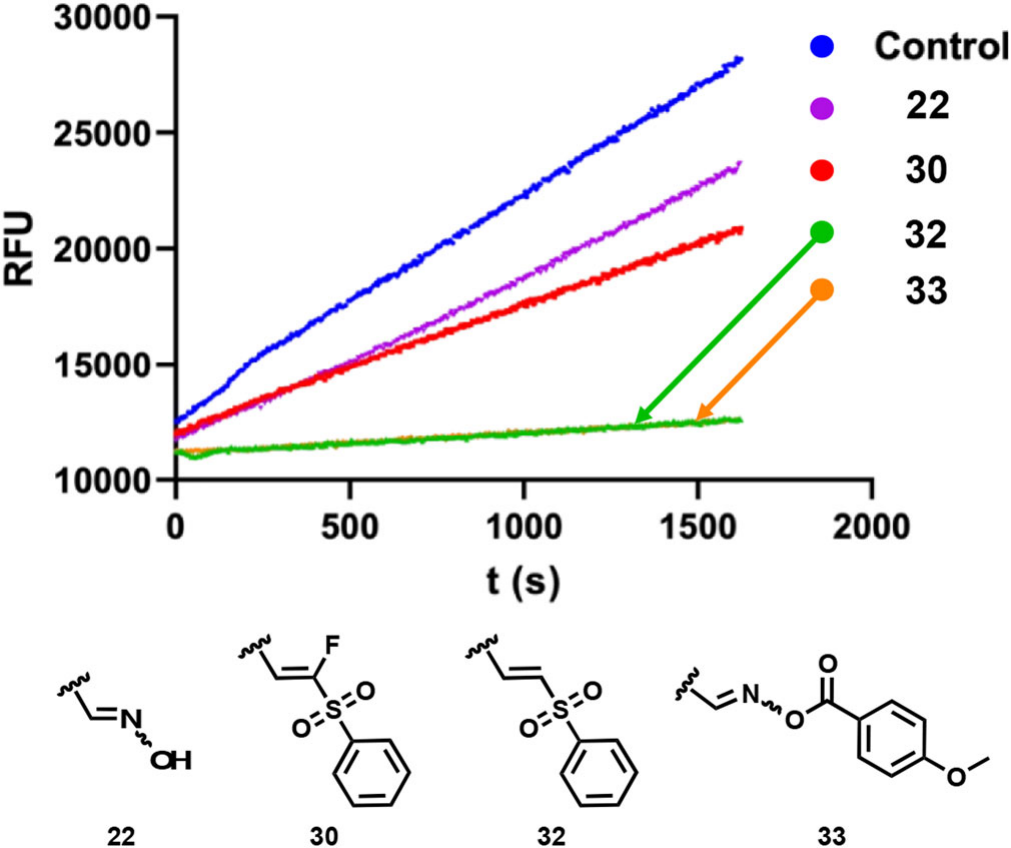
Jump‐dilution assay with inhibitors bearing distinct warheads against the WT SARS‐CoV‐2 Mpro. The inhibitor 22 bears an oxime as warhead, 30 a fluorine vinyl sulfone, 32 a vinyl sulphone, and 33 an oxime carbamate warhead. The control is composed of Mpro and substrate. The assay was conducted for 30 min.

This finding was obtained by using a jump‐dilution assay to assess the reversibility of the enzyme (M^pro^) when bound to its respective inhibitor (Figure 5). The compounds **22** and **30** displayed a reversible mechanism of inhibition. Notably, the obtained curves for these two compounds exhibit a similar behavior as the control, despite their lower slopes. This is expected, as a reversible inhibitor makes the enzyme less kinetically efficient. In addition, compounds **32** and **33** demonstrated an irreversible mechanism of inhibition, with very low residual enzyme activity.

The different reactivity between the vinyl sulphone **32** and the acylated oxime **33** could be attributed to the inherent distinct mechanism associated with each of these substances: the vinyl sulfone undergoes a nucleophilic addition, while the acylated oxime potentially experiences an addition‐elimination type of mechanism, in which the anisol carboxylate ring functions as a leaving group as the enzyme attacks the nitrogen atom. Evidently, the removal of this small fragment originally built into the molecule contributes to the entropic term of the covalent complex formation and, hence, results in the higher reactivity observed. This reactivity pattern would be comparable to a chloromethyl ketone warhead [28].

Irreversible warheads, due to their reactivity and formation of permanent covalent bonds with enzymes, are often criticized for potentially causing toxicity and permanently inactivating the targeted enzyme. Consequently, reversible inhibitors are generally preferred in drug development. However, covalent irreversible inhibitors can be beneficial in specific cases. For example, targeting a viral enzyme with no close human homologs, like M^pro^, guarantees definite selectivity and minimizes the risk of off‐target effects, making a highly reactive group ideal for terminating the enzyme's function without harming the host's enzymes.

**Figure 6:**
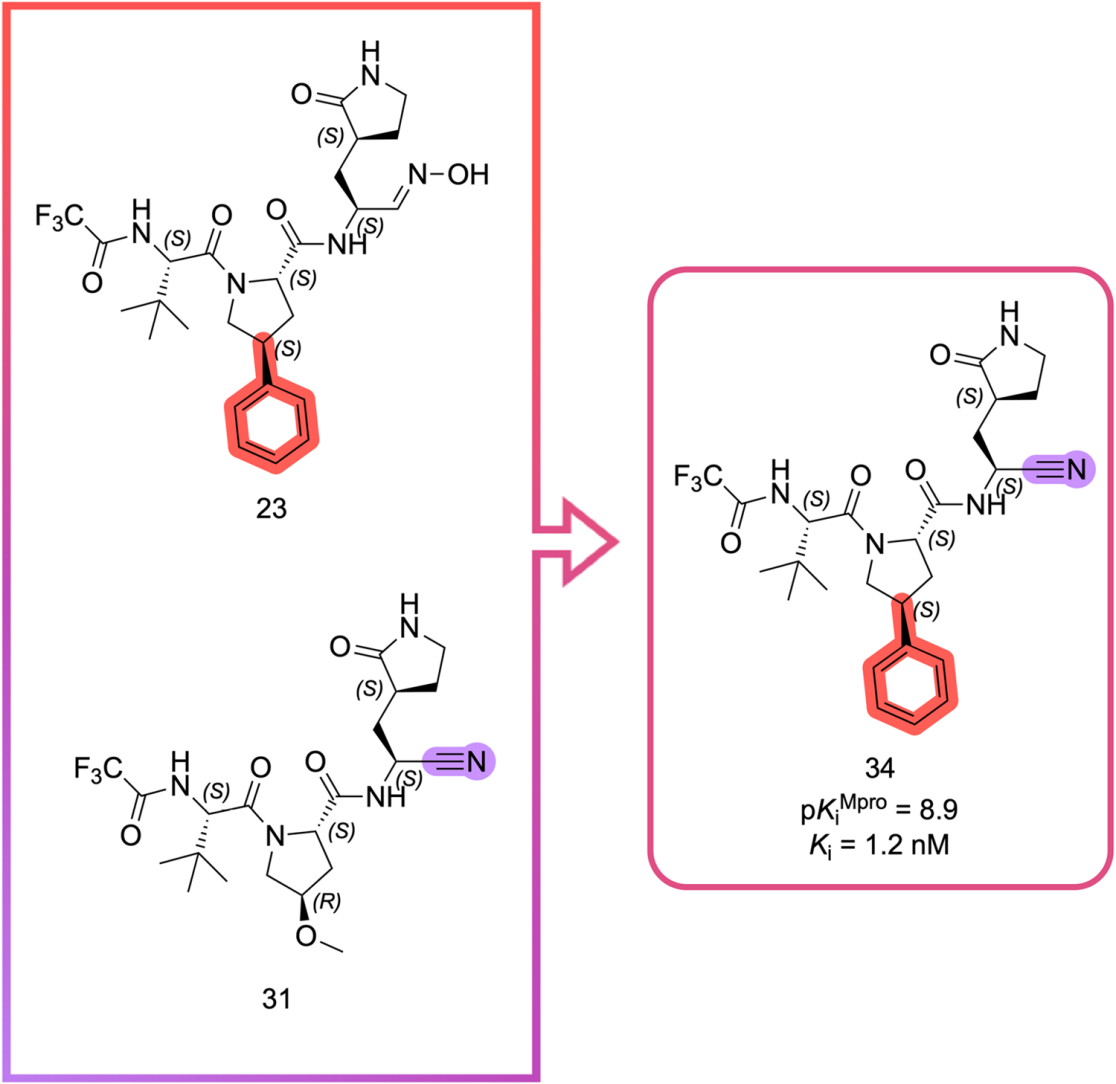
Fragment merging approach to create pharmacodynamic‐optimized hybrid compound **34**.

To produce the most optimized inhibitor, based on the results highlighted in MD simulations and Table 1 through a matched molecular pair analysis, we performed a fragment merging approach by keeping the P2 group found on compound **23**, the phenylproline. We also anchored the nitrile warhead, as seen on compound **31**, hence generating the hybrid compound. This can be seen from Figure 6. **34** stood out as one of the most promising compounds in this series, with a single‐digit *K*_i_ value, close to the picomolar range, with comparable potency to Pfizer's nirmatrelvir.

### 2.4 Selectivity Over Human Cysteine Proteases

All synthesized inhibitors were also biochemically evaluated for their inhibition activity against human cathepsins (CatL, S, and B). These have been proven to be strongly correlated to the progression of diverse diseases in the human body [29, 30]. None of the tripeptidyl inhibitors could inhibit these human targets.

This finding is rather unique in our research group, as some recent results indicated that some dipeptidyl compounds (not shown here) would be useful for dual targeting M^pro^ and CatL, for instance. Inhibiting both the viral protease and CatL, a host enzyme crucial for viral entry into cells, offers a more robust antiviral strategy. This dual inhibition could improve efficacy and create a synergistic effect, reducing the likelihood of resistance development [31, 32].

The intrinsic selectivity of M^pro^ for the proline‐based tripeptidyl compounds further qualifies the use of more reactive electrophilic warheads, such as the ones observed in **32** and **33**, as previously discussed. This finding is very singular: even though we employed hypersensitive electrophilic irreversible warheads, none of the human cathepsins tested had their activity inhibited by our compounds.

### 2.5 Metabolic Stability Studies

**Figure 7:**
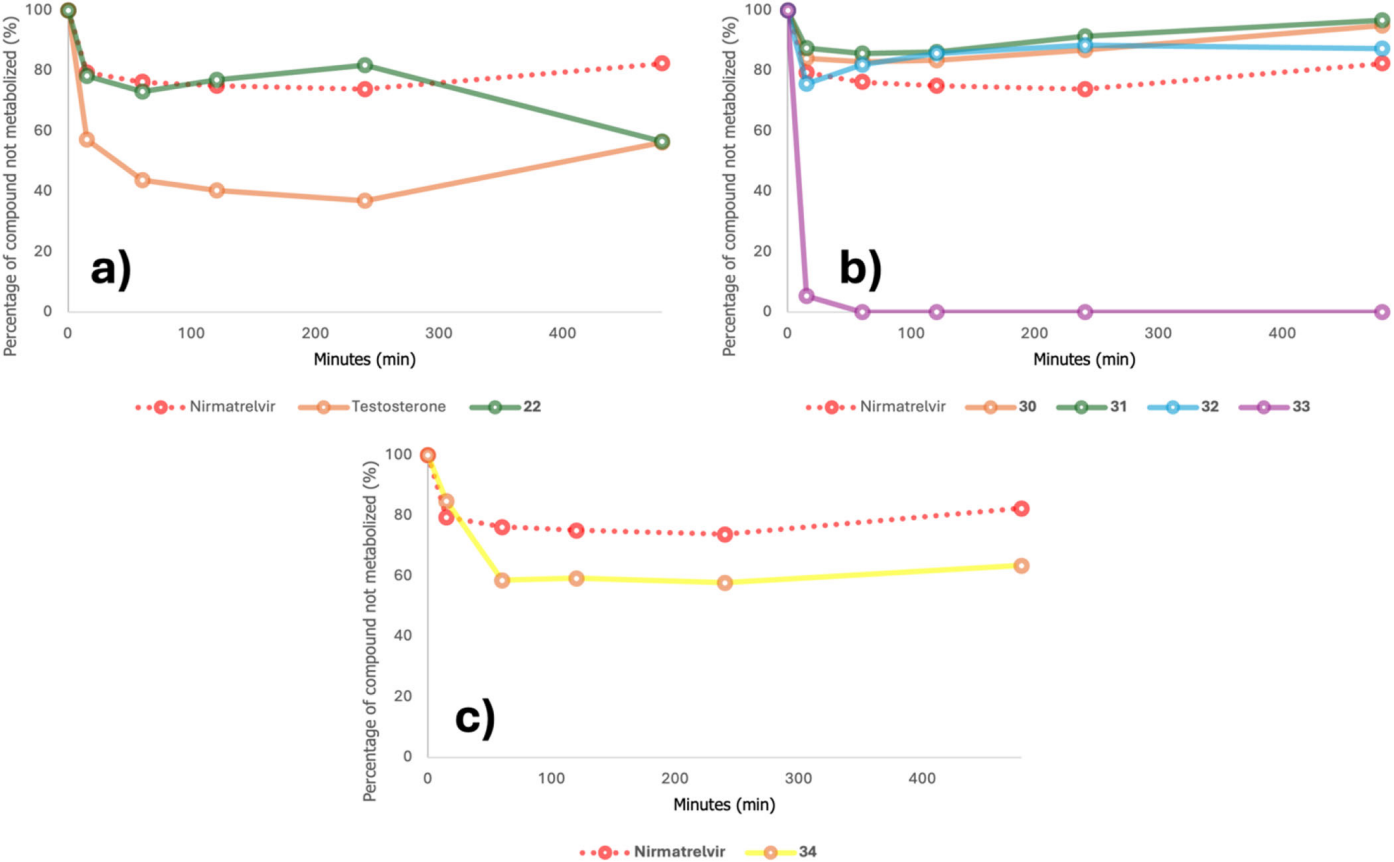
Metabolic activity data for selected compounds.

To assess whether our proposed molecules were more metabolically stable than nirmatrelvir, we performed a metabolic clearance test by using rat liver microsomes. Testosterone was utilized as a reference compound. As described elsewhere [33], each compound was incubated at 37°C for 480 min, and the diminishment of the parent compound was analyzed through HPLC with UV detection at each time interval. The results are presented in Figure 7.

While the reference compound testosterone is readily degraded by the liver microsomes, nirmatrelvir is relatively stable with ca. 80% remaining parent compound after 480 min (see Figure 7a). Our initial prototype compound **22** is slightly less stable with ca. 60% remaining after 480 min.

No relevant information was obtained from the P2 exchange strategy. Compounds **23**–**29** had their metabolic stability found to be close to 80% after the 480 min study and no specific substituent revealed a more stable profile.

Derivatives of **22** with modified electrophiles (**30**–**32**) also shown to be stable toward degradation by liver microsomes, with at least 80% compound intact even after 480 min (see Figure 7b).

In contrast, the acyl oxime electrophile in compound **33** displayed predictable fragmentation kinetics attributable to N–O bond lability. This structural instability correlates with rapid attenuation of UV absorbance signals during microsomal stability testing, confirming the anticipated release of acyl fragments from the parent molecule.

Lastly, compound **34** exhibited comparable stability to the initial prototype **22** (Figure 7c).

## 3 Conclusion

In this study, we successfully developed a series of compounds with a high inhibitory activity against SARS‐CoV‐2 M^pro^. Through an iterative process, we were able to identify compounds with different P2 and warheads that exhibited a high potency, as in **30** and **31**, demonstrating the effectiveness of our design strategies. The most potent compound **34** exhibited exceptional inhibitory activity, with a single‐digit nanomolar affinity (p*K*_i_ = 8.9).

Hence, **30**, the fluoro vinyl sulfone derivative, and **31**, the nitrile counterpart, are duly highlighted here, with similar affinities (p*K*_i_ = 8.1 and 8.8, respectively) and with strong metabolic stabilities, with close to 100% of the parent compound intact even after 8 h.

Balancing potency and metabolic stability is a significant challenge in drug discovery. This often involves trade‐offs, making the development of effective drug candidates a complex process. Testing for stability in rat liver microsomes very early in a drug discovery campaign allows for the identification of compounds that are highly susceptible to metabolic degradation. We could show that the acyl oxime of **33** is readily degraded by liver microsomes and thus should not be considered for future development. This reflects the ongoing need for careful optimization of both potency and stability in the development of therapeutics for COVID‐19. Nonetheless, through a combination of rational design, synthesis, and biochemical/biological evaluation, we present three novel lead structures **30**, **31**, and **34** with innovative P2 variations and electrophilic moieties. They show both strong inhibitory activity and stability toward liver microsomes in the same range as the approved nirmatrelvir.

## 4 Experimental

### 4.1 Chemistry

#### 4.1.1 General

Starting materials and solvents were acquired from commercial sources and directly utilized with no further purification. Amino acids were also purchased in their enantiomerically pure state. Macherey‐Nagel Polygram Sil G/UV254 polyester plates were employed to accompany the reaction progress and revealed under UV light at 254 nm in a Vilber Lourmat UV blackroom (CN‐6). When convenient, chemical revealing solutions were used, such as ninhydrin, phosphomolybdic acid, and potassium permanganate. Purification of intermediates was carried out in a Biotage Isolera One purification system using Biotage column (SNAP KP‐SIL) with silica gel (mesh 230–400, 60 Å) and UV monitoring at 254 and 280 nm. Purification of all final compounds was achieved using a preparative method on an ÄKTA pure 25 M from GE Healthcare (Germany) and a Dr. Maisch GmbH Reprospher C18, 5 µM, 120 mm × 16 mm HPLC column. The method employed is described herein:

- Water (eluent A) and acetonitrile (eluent B) gradient (10%–100%, 2.5–20 min, then 100%, 10 min) with a flow rate of 8 mL/min. Detection was achieved at 214, 254, and 280 nm.

After HPLC purification, the solvents were evaporated, and the compound was resolubilized in a water/acetonitrile mixture and then lyophilized in an Alpha 1‐2 LDplus freeze‐drier from Martin Christ, Germany. NMR spectra of final compounds were recorded on either a 300 or a 500 MHz Varian NMR instrument at room temperature. Chemical shifts (δ) are given in ppm. Coupling constants are given in hertz (Hz). Multiplicity is reported as usual. The mass spectra for the final compounds were obtained from a Brucker micrOTOF QII system using methanolic solutions. Final purity was checked on a Jasco HPLC system with a Jasco UV‐2070 Plus Intelligent UV/VIS Detector and with an RP‐18 column (ReproSil‐Pur‐ODS‐3, 5 µM, 50 mm × 2 mm). All final compounds present purity values ≥ 95%.

#### 4.1.2 General Synthetic Procedures

**General Procedure for Esterification (a):** Carboxylic acid 1 (2.00 mmol, 1.0 eq.) was dissolved in methanol (3 mL, 0.7 mol·L^−1^), and the flask was placed in an ice bath. Thionyl chloride (320 µL, 4.40 mmol, 2.2 eq.) was added dropwise, after which the reaction mixture was allowed to warm to room temperature and stirred overnight. The solvent was then evaporated, and the resulting amino ester 2 was used in the next step without further purification.

**General Procedure for the Synthesis of** ***O*** **‐Alkylated Proline Derivatives (b):** Sodium hydride (250 mg, 5.00 mmol, 2.5 eq.) was placed in a flask equipped with a stirring bar, nitrogen inlet, and rubber septum. After cooling the flask to 0°C under a nitrogen atmosphere, a solution of Boc‐protected hydroxyproline 3 (462 mg, 2.00 mmol, 1.0 eq.) in a dry THF/DMSO mixture (6.4 mL/0.4 mL, 16:1) was slowly added. The resulting heterogeneous mixture was stirred for an additional 30 min. Subsequently, a solution of the alkyl halide (2.5 eq.) in THF was added, and the reaction was allowed to warm to room temperature until complete conversion of the starting material. The reaction was quenched by adjusting the pH to 4 using a 10% aqueous potassium hydrogen sulfate solution. The crude product was then extracted with ethyl acetate, and the organic layer was washed with brine, dried over anhydrous magnesium sulfate, and concentrated under reduced pressure. Purification was performed using medium‐pressure liquid chromatography (MPLC).

**General Procedure for Peptide Coupling (c):** To a solution of amine 5 (0.50 mmol, 1.0 eq.) and carboxylic acid (0.60 mmol, 1.2 eq.) in DMF (5 mL), HATU (247 mg, 0.65 mmol, 1.3 eq.) was added, followed by the dropwise addition of DIPEA (0.3 mL, 1.50 mmol, 3.0 eq.). The reaction mixture was stirred at room temperature overnight (~16 h). The residue was then diluted with ethyl acetate (30 mL) and washed sequentially with saturated ammonium chloride solution (2 × 15 mL), saturated sodium hydrogen carbonate solution (2 × 15 mL), and brine (2 × 15 mL). The organic layer was dried over magnesium sulfate, filtered, and concentrated under vacuum. The crude product was purified by silica gel MPLC.

**General Procedure for the Hydrolysis of an Ester to a Carboxylic Acid (d):** A solution of ester 6 (0.28 mmol, 1.0 eq.) in tetrahydrofuran (0.7 mL, 0.40 mol·L^−1^) was treated with lithium hydroxide (20 mg, 0.83 mmol, 3.0 eq.) and water (0.4 mL, 2.0 mol·L^−1^). The reaction mixture was stirred at room temperature for 2 h before being diluted with water (10 mL) and ethyl acetate (15 mL), followed by treatment with 1 M aqueous sodium hydroxide solution (10 mL). The aqueous layer was washed with ethyl acetate (2 × 10 mL), and the combined organic layers were extracted three times with 1 M aqueous sodium hydroxide solution (3 × 10 mL). The aqueous layers were then acidified to pH 2 using concentrated HCl and extracted with ethyl acetate (3 × 15 mL). The organic layer was washed with brine (2 × 10 mL), dried over magnesium sulfate, and concentrated under vacuum to yield the pure carboxylic acid.

**General Procedure for Boc‐deprotection (e):** The Boc‐protected amine (0.94 mmol, 1.0 eq.) was dissolved in dry DCM (5 mL, 0.2 mol·L^−1^), and the reaction flask was placed in an ice bath. TFA (0.6 mL, 7.5 mmol, 8.0 eq.) was added dropwise, and the reaction mixture was stirred at room temperature. Reaction progress was monitored by TLC until complete conversion (~30 min) was achieved. The solvent was then removed under vacuum, and the crude deprotected amine was redissolved in dry DCM (5 mL). This process was repeated until no residual TFA was detected, yielding the TFA salt of amine 7, which was used in the next step without further purification.

**General Procedure for Insertion of the Trifluoroacetamide Group (f):** Trifluoroacetic anhydride (0.57 mmol, 1.3 eq.) was added dropwise to a suspension of amino acid 7 (0.47 mmol, 1.0 eq.) in DCM (5 mL, 0.1 mol·L^−1^). DIPEA (0.3 mL, 1.6 mmol, 3.4 eq.) was then added dropwise. The reaction mixture was stirred at room temperature for 3 h and then concentrated under vacuum.

**General Procedure for the Conversion of an Ester to an Amide (g):** A solution of ammonia in methanol (1.2 mL, 8.4 mmol, 7 N, 16.7 eq.) was added to Boc‐protected amino ester **10** (0.5 mmol, 1.0 eq.), and the mixture was stirred at room temperature for 48 h. The reaction was then concentrated under vacuum, yielding product **11**.

**General Procedure for the Reduction of an Ester to an Alcohol (h):** A solution of ester 10 (0.59 mmol, 1.0 eq.) in methanol (5 mL, 1.2 mol·L^−1^) was cooled to 0°C, and sodium borohydride (225 mg, 5.9 mmol, 10.0 eq.) was added in small portions. The reaction mixture was then stirred at room temperature until complete conversion of the starting material, guided by TLC (~1 h). The reaction was quenched with water (5 mL) and extracted with ethyl acetate (3 × 10 mL). The combined organic layers were washed with brine (2 × 5 mL), dried over magnesium sulfate, filtered, and concentrated under reduced pressure, yielding the crude product, which was used in the next step without further purification.

**General Procedure for the Oxidation of an Alcohol to an Aldehyde (i):** Dess–Martin periodinane (155 mg, 0.36 mmol, 1.2 eq.) was added to a solution of the alcohol (0.3 mmol, 1.0 eq.) in DCM (3 mL, 0.1 mol·L^−1^), and the mixture was stirred at room temperature for 2 h. The reaction mixture was then concentrated under vacuum and directly purified by MPLC, yielding aldehyde 13.

**General Procedure for Reductive Amination to Generate Oximes (j):** A solution of aldehyde 13 (0.2 mmol, 1.0 eq.) in ethanol (2 mL, 0.1 mol·L^−1^) was treated with sodium hydrogen carbonate (35 mg, 0.4 mmol, 2.0 eq.) and hydroxylamine hydrochloride (28 mg, 0.4 mmol, 2.0 eq.). The mixture was stirred at room temperature overnight, then washed with water (2 × 10 mL), concentrated under vacuum, and purified by MPLC, yielding oxime 14.

**General Procedure for the Synthesis of a Phosphonate:** A solution of n‐butyllithium (24 mL, 60 mmol, 2.5 M in hexanes, 2.22 eq.) was added dropwise to a solution of methyl phenyl sulfone (4.24 g, 27 mmol, 1.0 eq.) in dry THF (25 mL, 1.1 mol·L^−1^) at 0°C. After stirring for 30 min, diethyl chlorophosphate (4.7 mL, 32 mmol, 1.2 eq.) was added dropwise. The reaction was quenched after 1 h with ammonium chloride (20 mL), and the organic volatiles were removed under vacuum. The residue was then extracted with dichloromethane (3 × 15 mL), and the combined organic layers were dried over magnesium sulfate, filtered, concentrated, and purified by MPLC. The product was obtained as a colorless oil (6.87 g, 87% yield).

**General Procedure for the Fluorination of a Phosphonate:** The phosphonate (2.9 g, 10 mmol, 1.0 eq.) was dissolved in dry THF (15 mL, 0.7 mol·L^−1^) and cooled to −78°C. KHMDS (12.5 mL, 12.5 mmol, 1 M in THF, 1.25 eq.) was then added dropwise, and the reaction was stirred at this temperature for 30 min. Subsequently, Selectfluor (5.3 g, 15 mmol, 1.5 eq.) was dissolved in THF (10 mL, 1.5 mol·L^−1^) and added to the reaction flask. The cooling bath was removed and stirring continued for 3 h at room temperature. The reaction was quenched with a saturated ammonium chloride solution (15 mL), and volatiles were removed under vacuum. The residue was extracted with dichloromethane (3 × 15 mL), and the combined organic layers were washed with saturated sodium hydrogen carbonate solution (2 × 10 mL) and brine (2 × 10 mL), dried over magnesium sulfate, concentrated under reduced pressure, and purified by MPLC. The product was obtained as a colorless oil (2.26 g, 73% yield).

**General Procedure for the Horner–Wadsworth–Emmons Olefination Reaction (k):** The phosphonate or fluorophosphonate (0.9 mmol, 1.0 eq.) was dissolved in dry THF (6 mL, 0.15 mol·L^−1^) and cooled to −78°C. LHMDS (0.9 mL, 0.9 mmol, 1 M in THF, 1.0 eq.) was added dropwise, and the reaction mixture was stirred at this temperature for 30 min. Aldehyde 13 (0.9 mmol, 1.0 eq.) was then added in one portion, after which the cooling bath was removed, and stirring continued for 2 h. The reaction was diluted with ethyl acetate (15 mL), and the organic phase was washed with water (2 × 10 mL) and brine (2 × 10 mL). The solvent was evaporated under reduced pressure, yielding the crude product—compound **18** when using the phosphonate and compound **20** when using the fluorophosphonate—as a mixture of E/Z isomers. The product was isolated by MPLC.

#### 4.1.3 Characterization of Final Compounds

**(2S,4R)−1‐{(S)−3,3‐Dimethyl‐2‐(2,2,2‐Trifluoroacetamido)butanoyl}‐N‐[(S)−1‐(Hydroxyimino)−3‐[(S)−2‐Oxopyrrolidin‐3‐yl]Propan‐2‐yl]−4‐Methoxypyrrolidine‐2‐Carboxamide (22).**

Obtained as a white solid (123 mg, 52% yield over six steps). ^1^H NMR (300 MHz, Acetone‐d6) *δ* 10.02 (s, 1H, OH), 7.70 (d, *J* = 9.0 Hz, 1H, NH), 7.36 (d, *J* = 5.2 Hz, 1H, NH), 6.93 (d, *J *= 6.0 Hz, 1H, NH), 4.78–4.62 (m, 1H, CH), 4.41 (dd, *J* = 9.1, 7.5 Hz, 1H, CH), 4.12 (n, *J* = 3.8, 1.6 Hz, 1H, CH), 4.00 (dd, *J *= 11.4, 1.6 Hz, 1H, CH_2_), 3.77 (ddd, *J *= 11.2, 3.9, 1.9 Hz, 1H, CH_2_), 3.28 (s, 3H, OCH_3_), 3.19 (ddd, *J* = 14.5, 11.8, 7.3 Hz, 2H, CH_2_), 2.75–2.60 (m, 1H, CH_2_), 2.50–2.37 (m, 1H, CH_2_), 2.32 (m, *J* = 13.2, 7.6, 1.6 Hz, 1H, CH_2_), 2.22–2.11 (m, 1H, CH_2_), 1.87–1.58 (m, 2H, CH_2_), 1.53 (ddd, *J *= 14.3, 11.1, 3.6 Hz, 1H, CH_2_), 1.08 (s, 9 H, 3 CH_3_). ^13^C NMR (75 MHz, Acetone‐d6) *δ* 180.24 (CO), 172.04 (CO), 169.27 (CO), 152.13 (CN), 150.73 (CO), 80.03 (CO), 60.40 (CH), 58.74 (CH), 56.35 (CH_3_), 54.04 (CH), 47.43 (CH_2_), 44.43 (CH), 40.60 (quat. C), 38.19 (CH_2_), 36.79 (CH_2_), 35.65 (CH_2_), 34.18 (CH), 26.72 (3 CH_3_). HRMS (ESI) *m/z*: [M + Na]^+^ calculated for C_21_H_32_F_3_N_5_O_6_: 530.2197; found: 530.2207.

**(2S,4S)−1‐{(S)−3,3‐Dimethyl‐2‐(2,2,2‐Trifluoroacetamido)Butanoyl}‐N‐[(S)−1‐(Hydroxyimino)−3‐[(S)−2‐Oxopyrrolidin‐3‐yl]Propan‐2‐yl]−4‐Phenylpyrrolidine‐2‐Carboxamide (23).**

Obtained as a white solid (177 mg, 48% yield over six steps). ^1^H NMR (300 MHz, Acetone‐d6) *δ* 9.93 (s, 1H, OH), 8.10 (d, *J* = 8.6 Hz, 1H, NH), 7.64 (d, *J* = 9.0 Hz, 1H, NH), 7.39 (d, *J* = 5.1 Hz, 1H, NH), 7.36–7.30 (m, 4H, Ar‐H), 7.24 (m, *J* = 8.1, 3.9, 2.3 Hz, 1H, Ar‐H), 6.78 (d, *J* = 14.1 Hz, 1H, CH), 4.79 (d, *J* = 9.3 Hz, 1H, CH), 4.57 (m, *J* = 12.0, 7.1, 4.3 Hz, 1H, CH), 4.38–4.24 (m, 1H, CH), 3.77 (m, *J* = 7.7, 7.0 Hz, 1H, CH), 3.35–3.14 (m, 1H, CH_2_), 2.68–2.48 (m, 1H, CH_2_), 2.51–2.35 (m, 1H, CH_2_), 2.39–2.27 (m, 1H, CH_2_), 2.19–2.06 (m, 1H, CH_2_), 2.04–1.85 (m, 1H, CH_2_), 1.79 (ddd, *J* = 20.1, 9.9, 2.6 Hz, 1H, CH_2_), 1.75–1.49 (m, 1H, CH_2_), 1.11 (s, 9H, 3 CH_3_), 1.01 (d, *J* = 4.8 Hz, 1H, CH). ^13^C NMR (75 MHz, Acetone‐d6) *δ* 179.23 (CO), 172.29 (2 CO), 150.78 (CN), 141.66 (quat. C), 129.46 (Ar‐H), 128.01 (Ar‐H), 127.75 (Ar‐H), 61.20 (CH), 58.51 (CH), 54.88 (CH), 47.65 (CH_2_), 43.78 (CH_2_), 40.55 (quat. C), 38.29 (CH_2_), 37.17 (CH_2_), 36.60 (CH_2_), 35.58 (CH_2_), 26.72 (3 CH_3_). HRMS (ESI) *m/z*: [M + Na]^+^ calculated for C_26_H_34_F_3_N_5_O_5_: 576.2404; found: 576.2418.

**(2S,4R)−4‐(Allyloxy)−1‐{(S)−3,3‐Dimethyl‐2‐(2,2,2‐Trifluoroacetamido)Butanoyl}‐N‐[(S)−1‐(Hydroxyimino)−3‐[(S)−2‐Oxopyrrolidin‐3‐yl]Propan‐2‐yl]Pyrrolidine‐2‐Carboxamide (24).**

Obtained as a white solid (103 mg, 21% yield over seven steps). ^1^H NMR (300 MHz, Acetone‐d6) *δ* 10.02 (s, 1H, OH), 8.23 (d, *J *= 9.0 Hz, 1H, NH), 7.69 (d, *J *= 9.0 Hz, 1H, NH), 7.36 (d, *J *= 5.2 Hz, 1H), NH, 5.88 (ddt, *J *= 17.3, 10.6, 5.3 Hz, 1H, CH), 5.34–5.18 (m, 1H, CH_2_), 5.09 (dq, *J* = 10.4, 1.6 Hz, 1H, CH_2_), 4.77–4.71 (m, 1H, CH), 4.51–4.37 (m, 1H, CH), 4.29 (m, *J* = 4.2, 1.8 Hz, 1H, CH), 4.02 (dt, *J* = 5.3, 1.6 Hz, 2H, CH_2_), 3.99–3.93 (m, 1H, CH_2_), 3.80 (ddd, *J *= 11.2, 4.0, 2.2 Hz, 1H, CH_2_), 3.32–3.12 (m, 2H, CH_2_), 2.67 (m, *J *= 11.1, 8.3, 3.6 Hz, 1H, CH_2_), 2.47–2.36 (m, 1H, CH_2_), 2.31 (m, *J* = 13.2, 7.6, 1.9 Hz, 1H, CH_2_), 1.85–1.62 (m, 1H, CH_2_), 1.07 (s, 9H, 3 CH_3_), 0.97 (d, *J* = 3.1 Hz, 1H, CH). ^13^C NMR (75 MHz, Acetone‐d6) *δ* 180.19 (CO), 172.20 (CO), 172.02 (CO), 169.28 (CO), 150.71 (CN), 135.99 (CH), 116.64 (CH_2_), 78.06 (CH), 70.09 (CH), 60.47 (CH), 58.71 (CH), 54.46 (CH), 47.45 (CH_2_), 44.45 (CH_2_), 40.58 (quat. C), 38.18 (CH_2_), 36.83 (CH_2_), 36.09 (CH_2_), 35.53 (CH_2_), 34.16 (CH_2_), 26.72 (3 CH_3_). HRMS (ESI) *m/z*: [M + Na]^+^ calculated for C_23_H_34_F_3_N_5_O_6_: 556.2353; found: 556.2373.

**(2S,4R)−1‐{(S)−3,3‐Dimethyl‐2‐(2,2,2‐Trifluoroacetamido)Butanoyl}‐N‐[(S)−1‐(Hydroxyimino)−3‐[(S)−2‐Oxopyrrolidin‐3‐yl]Propan‐2‐yl]−4‐(Prop‐2‐yn‐1‐Yloxy)Pyrrolidine‐2‐Carboxamide (25).**

Obtained as a white solid (97 mg, 24% yield over seven steps). ^1^H NMR (300 MHz, Acetone‐d6) *δ* 10.01 (s, 1H, OH), 8.22 (d, *J* = 10.1 Hz, 1H, NH), 7.70 (d, *J* = 9.1 Hz, 1H, NH), 7.36 (d, *J* = 5.2 Hz, 1H, NH), 6.87 (d, *J* = 6.7 Hz, 1H, CH), 4.71 (m, *J* = 12.4, 8.9, 4.5 Hz, 2H, 2 CH), 4.54–4.37 (m, 2H, CH_2_), 4.22 (d, *J *= 2.5 Hz, 2H, CH_2_), 4.04 (dd, *J *= 11.4, 1.7 Hz, 2H, CH_2_), 3.84 (m, *J* = 11.4, 4.0, 1.8 Hz, 1H, CH), 3.37–3.12 (m, 2H, CH_2_), 2.95 (t, *J *= 2.4 Hz, 1H, CH), 2.67 (tdd, *J* = 10.9, 8.3, 3.6 Hz, 1H, CH_2_), 2.48–2.29 (m, 2H, CH_2_), 2.19–2.10 (m, 1H, CH_2_), 2.10–2.07 (m, 1H, CH_2_), 1.86–1.59 (m, 1H, CH_2_), 1.53 (ddd, *J* = 14.4, 11.1, 3.6 Hz, 1H, CH_2_), 1.08 (s, 9H, 3 CH_3_), 0.97 (d, *J *= 3.1 Hz, 1H, CH_2_). ^13^C NMR (75 MHz, Acetone‐d6) *δ* 180.15 (CO), 171.93 (CO), 169.23 (CO), 152.14 (CN), 150.70 (CO), 80.59 (CH), 77.74 (CC), 76.17 (CC), 60.40 (CH), 58.78 (CH), 56.46 (CH_2_), 54.19 (CH), 47.47 (CH_2_), 44.45 (CH_2_), 40.57 (quat. C), 38.39 (CH_2_), 38.17 (CH_2_), 36.89 (CH_2_), 35.82 (CH_2_), 35.53 (CH_2_), 34.17 (CH_2_), 26.72 (3 CH_3_). HRMS (ESI) *m/z*: [M + Na]^+^ calculated for C_23_H_32_F_3_N_5_O_6_: 554.2197; found: 554.2212.

**(2S,4R)−1‐{(S)−3,3‐Dimethyl‐2‐(2,2,2‐Trifluoroacetamido)Butanoyl}−4‐Ethoxy‐N‐[(S)−1‐(Hydroxyimino)−3‐[(S)−2‐Oxopyrrolidin‐3‐yl]Propan‐2‐yl]Pyrrolidine‐2‐Carboxamide (26).**

Obtained as a white solid (141 mg, 20% yield over seven steps). ^1^H NMR (300 MHz, Acetone‐d6) *δ* 9.97 (s, 1H, OH), 8.22 (d, *J* = 9.2 Hz, 1H, NH), 7.65 (d, *J *= 9.1 Hz, 1H, NH), 7.36 (d, *J *= 5.2 Hz, 1H, NH), 6.83 (d, *J* = 9.1 Hz, 1H, CH), 4.73 (d, *J* = 9.2 Hz, 1H, CH), 4.41 (ddd, *J* = 10.2, 8.5, 4.6 Hz, 1H, CH), 4.22 (dq, *J* = 4.7, 2.2 Hz, 1H, CH), 3.98–3.91 (m, 1H, CH_2_), 3.79 (ddd, *J* = 11.2, 4.0, 1.8 Hz, 1H, CH_2_), 3.49 (qt, *J* = 6.5, 2.0 Hz, 2H, CH_2_), 3.39–3.13 (m, 2H, CH_2_), 2.73–2.59 (m, 1H, CH_2_), 2.55–2.34 (m, 1H, CH_2_), 2.34–2.19 (m, 1H, CH_2_), 2.01–1.83 (m, 1H, CH_2_), 1.83–1.69 (m, 1H, CH_2_), 1.14–1.09 (m, 3H, CH_3_), 1.07 (d, *J* = 2.2 Hz, 9H, 3 CH_3_), 1.04 (d, *J* = 2.6 Hz, 2H, CH_2_), 1.00–0.96 (m, 1H, CH_2_). ^13^C NMR (75 MHz, Acetone‐d6) *δ* 180.05 (CO), 172.04 (CO), 169.24 (CO), 152.25 (CN), 150.74 (CO), 129.04 (CF), 78.21 (CH), 64.60 (CH_2_), 60.46 (CH), 58.70 (CH), 54.45 (CH), 47.47 (CH_2_), 40.55 (quat. C), 38.17 (CH_2_), 36.79 (CH_2_), 36.12 (CH_2_), 35.57 (CH_2_), 26.72 (3 CH_3_), 15.62 (CH_3_). HRMS (ESI) *m/z*: [M + Na]^+^ calculated for C_22_H_34_F_3_N_5_O_6_: 544.2353; found: 544.2359.

**(2S,4S)−4‐Cyclohexyl‐1‐{(S)−3,3‐Dimethyl‐2‐(2,2,2‐Trifluoroacetamido)Butanoyl}‐N‐[(S)−1‐(Hydroxyimino)−3‐[(S)−2‐Oxopyrrolidin‐3‐yl]Propan‐2‐yl]Pyrrolidine‐2‐Carboxamide (27).**

Obtained as a white solid (110 mg, 42% yield over six steps). ^1^H NMR (300 MHz, Acetone‐d6) *δ* 7.37 (d, *J* = 5.1 Hz, 1H, NH), 4.75 (d, *J* = 6.3 Hz, 1H, CH), 4.72–4.64 (m, 1H, CH), 4.56–4.42 (m, 1H, CH), 4.03 (ddd, *J* = 12.0, 9.8, 7.8 Hz, 1H, CH_2_), 3.43–3.29 (m, 2H, CH_2_), 3.29–3.13 (m, 2H, CH_2_), 2.63–2.48 (m, 1H, CH_2_), 2.48–2.34 (m, 1H, CH_2_), 2.34–2.17 (m, 1H, CH_2_), 2.10– 2.07 (m, 2H, CH_2_), 2.01–1.92 (m, 1H, CH_2_), 1.90–1.78 (m, 1H, CH_2_), 1.78–1.66 (m, 6H, CH_2_), 1.66–1.57 (m, 2H, CH_2_), 1.57–1.45 (m, 1H, CH_2_), 1.21 (tt, *J *= 13.2, 7.1 Hz, 4H, CH_2_), 1.08 (s, 9H, 3 CH_3_). ^13^C NMR (75 MHz, Acetone‐d6) *δ* 180.06 (CO), 172.44–171.96 (m, 2 CO), 169.31 (CO), 169.01 (CO), 152.08 (CN), 151.12 (CF), 150.89 (CF), 150.62 (CF), 61.38 (CH), 61.27 (CH), 61.19 (CH), 52.83 (CH), 52.27 (CH_2_), 47.39 (CH_2_), 44.82 (CH_2_), 44.40 (CH_2_), 42.17 (CH_2_), 42.03 (CH_2_), 40.49 (quat. C), 40.02 (CH_2_), 38.56 (CH_2_), 38.33 (CH_2_), 38.04 (CH_2_), 36.55 (CH_2_), 36.49 (CH_2_), 35.56 (CH_2_), 34.12 (CH_2_), 34.03 (CH_2_), 33.98 (CH_2_), 33.79 (CH_2_), 33.62 (CH_2_), 32.48 (CH), 31.89 (CH_2_), 27.01 (CH_2_), 26.71 (CH_3_). HRMS (ESI) *m/z*: [M + Na]^+^ calculated for C_26_H_40_F_3_N_5_O_5_: 582.2874; found: 582.2887.

**(2S,4R)−4‐Acetamido‐1‐{(S)−3,3‐Dimethyl‐2‐(2,2,2‐Trifluoroacetamido)Butanoyl}‐N‐[(S)−1‐(Hydroxyimino)−3‐[(S)−2‐Oxopyrrolidin‐3‐yl]Propan‐2‐yl]Pyrrolidine‐2‐Carboxamide (28).**

Obtained as a white solid (122 mg, 48% yield over five steps). ^1^H NMR (300 MHz, acetone) *δ* 9.93 (d, *J* = 3.5 Hz, 1H, OH), 8.07 (s, 1H, NH), 7.61 (d, *J* = 8.9 Hz, 1H, NH), 7.43–7.27 (m, 1H, NH), 6.75 (d, *J* = 12.5 Hz, 1H, CH), 4.68 (dd, *J* = 9.2, 4.7 Hz, 2H, 2 CH), 4.57 (d, *J* = 8.3 Hz, 1H, CH), 4.54–4.47 (m, 1H, CH), 4.44 (dd, *J* = 9.1, 4.6 Hz, 1H, CH), 4.05–3.93 (m, 1H, CH_2_), 3.72 (m, *J* = 10.4, 4.5 Hz, 1H, CH_2_), 3.30 (m, 2H, CH_2_), 3.22 (dd, *J* = 16.6, 7.5 Hz, 1H, CH_2_), 2.64–2.48 (m, 1H CH_2_), 2.40 (m, 2H, CH_2_), 2.19 (t, *J* = 6.6 Hz, 2H, CH_2_), 1.88–1.76 (m, 4H, 2 CH_2_), 1.08 (d, *J* = 1.3 Hz, 9H, 3 CH_3_), 1.04 (d, *J* = 1.3 Hz, 4H, 2 CH_2_). HRMS (ESI) *m/z*: [M + Na]^+^ calculated for C_22_H_33_F_3_N_6_O_6_: 557.2306; found: 557.2312.

**(2S,4R)−4‐(Benzyloxy)−1‐{(S)−3,3‐Dimethyl‐2‐(2,2,2‐Trifluoroacetamido)Butanoyl}‐N‐[(S)−1‐(Hydroxyimino)−3‐[(S)−2‐Oxopyrrolidin‐3‐yl]Propan‐2‐yl]Pyrrolidine‐2‐Carboxamide (29).**

Obtained as a white solid (132 mg, 25% yield over seven steps). ^1^H NMR (300 MHz, acetone) *δ* 9.77 (s, 1H, OH), 9.07 (d, *J* = 8.7 Hz, 1H, NH), 7.38–7.31 (m, 5H, Ar‐H), 7.27 (m, *J* = 8.9, 2.6 Hz, 2H, NH), 7.15 (d, *J* = 5.3 Hz, 1H, NH), 4.71–4.63 (m, 1H, CH), 4.59–4.49 (m, 3H, 3 CH), 4.49–4.39 (m, 1H, CH), 4.38–4.30 (m, 1H, CH), 4.28 (m, *J* = 5.0, 2.4 Hz, 1H, CH), 3.96 (dt, *J* = 10.4, 5.1 Hz, 1H, CH_2_), 3.91–3.78 (m, 1H, CH_2_), 3.28 (dt, *J* = 18.0, 8.4 Hz, 2H, CH_2_), 2.49 (dd, *J* = 9.0, 5.4 Hz, 1H, CH_2_), 2.43–2.30 (m, 2H, CH_2_), 2.26 (dt, *J* = 13.6, 3.7 Hz, 1H, CH_2_), 1.97–1.84 (m, 1H, CH_2_), 1.84–1.66 (m, 1H), 1.60 (ddd, *J* = 14.3, 7.8, 4.1 Hz, 1H, CH_2_), 1.08 (s, 2H, CH_2_), 1.04 (d, *J* = 3.2 Hz, 9H, 3 CH_3_). ^13^C NMR (75 MHz, acetone) *δ* 171.63 (CO), 169.40 (CO), 151.04 (CN), 129.22 (Ar‐C), 129.17 (Ar‐C), 129.10 (Ar‐C), 128.70 (Ar‐C), 128.64 (Ar‐C), 128.46 (Ar‐C), 128.39 (Ar‐C), 77.72 (CO), 71.25 (CH_2_), 61.23 (CH), 58.65 (CH), 53.95 (CH), 48.61 (CH_2_), 40.97 (quat. C), 39.17 (CH_2_), 38.58 (CH_2_), 36.36 (CH_2_), 35.18 (CH_2_), 26.72 (3 CH_3_). HRMS (ESI) *m/z*: [M + Na]^+^ calculated for C_27_H_36_F_3_N_5_O_6_: 606.2510; found: 606.2515.

**(2S,4R)−1‐{(S)−3,3‐Dimethyl‐2‐(2,2,2‐Trifluoroacetamido)Butanoyl}‐N‐[(S,E)−4‐Fluoro‐1‐((S)−2‐Oxopyrrolidin‐3‐yl)−4‐(Phenylsulfonyl)but‐3‐en‐2‐yl]−4‐Methoxypyrrolidine‐2‐Carboxamide (30).**

Obtained as a white solid (201 mg, 55% yield over five steps). ^1^H NMR ((300 MHz, Acetone‐d6) *δ* 8.21 (dd, *J* = 8.5, 1.4 Hz, 1H, NH), 8.17–8.12 (m, 1H, Ar‐H), 8.06–7.92 (m, 2H, Ar‐H), 7.87–7.78 (m, 1H, Ar‐H), 7.78–7.66 (m, 2H‐ Ar‐H), 6.87 (d, *J *= 14.0 Hz, 1H, NH), 6.38 (dd, *J* = 33.1, 8.3 Hz, 1H, CH, *E* isomer), 4.78–4.69 (m, 1H, CH), 4.34 (dd, *J* = 9.2, 7.5 Hz, 1H, CH_2_), 4.09 (qd, *J* = 4.6, 3.2, 2.4 Hz, 1H, CH_2_), 4.05–3.94 (m, 1H CH_2_), 3.77 (ddd, *J* = 15.3, 11.3, 3.9 Hz, 1H, CH_2_), 3.39–3.30 (m, 1H CH_2_), 3.28 (s, 3H, CH_3_), 3.26–3.13 (m, 1H, CH_2_), 2.69 (dddd, *J* = 20.5, 12.7, 10.2, 5.2 Hz, 1H, CH_2_), 2.58–2.38 (m, 1H, CH_2_), 2.37–2.25 (m, 1H, CH_2_), 2.25–2.10 (m, 1H, CH_2_), 1.96 (m, *J* = 17.5, 11.0, 3.9 Hz, 1H, CH_2_), 1.80–1.61 (m, 1H, CH_2_), 1.50 (m, J = 24.3, 14.0, 10.2, 3.8 Hz, 1H, CH_2_), 1.18–1.01 (m, 9H, 3 CH_3_), 0.96 (d, *J* = 10.3 Hz, 1H CH_2_). ^13^C NMR (75 MHz, Acetone‐d6) *δ* 179.64 (CO), 172.03 (CO), 169.25 (CO), 135.77 (d, *J* = 10.7 Hz, CF), 130.66 (d, *J* = 5.1 Hz, Ar‐C), 129.57 (d, *J* = 6.3 Hz, Ar‐C), 129.33 (Ar‐C), 120.13 (d, *J* = 4.9 Hz, Ar‐C), 80.00 (CO), 60.42 (d, *J *= 7.6 Hz, CH), 58.74 (CH), 56.35 (OCH_3_), 53.92 (d, *J* = 10.9 Hz, CH), 44.35 (d, *J *= 6.4 Hz, CH_2_), 43.86 (CH_2_), 40.54 (t, *J* = 9.4 Hz, quat. C), 38.33 (t, *J* = 17.8 Hz, CH_2_), 36.76 (CH_2_), 35.59 (d, *J* = 10.7 Hz, CH_2_), 26.72 (d, *J* = 3.3 Hz, 3 CH_3_). HRMS (ESI) *m/z*: [M + Na]^+^ calculated for C_28_H_36_F_4_N_4_O_7_S: 671.2133; found: 671.2149.

**(2S,4R)‐N‐[(S)−1‐Cyano‐2‐[(S)−2‐Oxopyrrolidin‐3‐yl]Ethyl]−1‐{(S)−3,3‐Dimethyl‐2‐(2,2,2‐Trifluoroacetamido)Butanoyl}−4‐Methoxypyrrolidine‐2‐Carboxamide (31).**

Obtained as a white solid (176 mg, 52% yield over five steps). ^1^H NMR (300 MHz, acetone) δ 8.35 (d, *J* = 8.3 Hz, 1H, NH), 8.16 (d, *J* = 9.0 Hz, 1H, NH), 6.92 (s, 1H, NH), 5.09 (ddd, *J* = 11.4, 8.4, 4.7 Hz, 1H, CH), 4.73 (d, *J* = 9.2 Hz, 1H, CH), 4.38 (dd, *J* = 9.3, 7.6 Hz, 1H, CH), 4.14 (s broad, 1H, NH), 4.02 (d, *J* = 11.3 Hz, 1H, CH_2_), 3.78 (dd, *J* = 11.3, 3.8 Hz, 1H, CH_2_), 3.30 (d, *J* = 2.3 Hz, 3H, CH_3_), 3.27–3.17 (m, 2H, CH_2_), 2.66 (ddt, *J* = 15.0, 10.0, 4.9 Hz, 1H, CH_2_), 2.43–2.24 (m, 3H, CH_2_), 1.94–1.75 (m, 2H, CH_2_), 1.07 (s, 9H, 3 CH_3_), 0.95 (s, 1H, CH_2_). ^13^C NMR (75 MHz, acetone) *δ* 178.91 (CO), 172.35 (CO), 169.38 (CO), 119.97 (CN), 80.02 (CO), 60.12 (CH), 58.69 (CH), 56.37 (CH_3_), 54.00 (CH), 40.56 (quat. C), 39.13 (CH_2_), 37.96 (CH_2_), 36.72 (CH_2_), 35.74 (CH_2_), 35.47 (CH_2_), 28.61 (CH_2_), 26.67 (3 CH_3_). HRMS (ESI) *m/z*: [M + Na]^+^ calculated for C_21_H_30_F_3_N_5_O_5_: 512.2091; found: 512.2085.

**(2S,4R)−1‐{(S)−3,3‐Dimethyl‐2‐(2,2,2‐Trifluoroacetamido)butanoyl}−4‐Methoxy‐N‐[(S,E)−1‐((S)−2‐Oxopyrrolidin‐3‐yl)−4‐(Phenylsulfonyl)but‐3‐en‐2‐yl]Pyrrolidine‐2‐Carboxamide (32).**

Obtained as a white solid (188 mg, 59% yield over five steps). ^1^H NMR (300 MHz, Acetone‐d6) *δ* 8.20 (d, *J *= 9.3 Hz, 1H, NH), 7.94–7.83 (m, 3H, Ar‐H), 7.76–7.68 (m, 1H, Ar‐H), 7.68–7.59 (m, 2H, Ar‐H), 6.97 (dd, *J* = 15.1, 4.3 Hz, 1H, *E* isomer, CH), 6.81 (s, 1H, NH), 6.70 (dd, *J* = 15.1, 1.8 Hz, 1H, *E* isomer, CH), 4.75–4.68 (m, 1H, CH), 4.36 (dd, *J *= 9.3, 7.5 Hz, 1H, CH), 4.08 (dd, *J* = 4.2, 2.2 Hz, 1H, CH), 3.97 (d, *J* = 11.0 Hz, 1H, CH_2_), 3.77 (dd, *J* = 11.2, 3.9 Hz, 1H, CH_2_), 3.26 (s, 3H, CH_3_), 3.25–3.14 (m, 1H, CH_2_), 2.67 (tdd, *J* = 10.2, 8.4, 4.3 Hz, 1H, CH_2_), 2.35 (ddq, *J *= 11.5, 7.9, 1.8 Hz, 2H, CH_2_), 2.02–1.90 (m, 2H, CH_2_), 1.90–1.82 (m, 1H, CH_2_), 1.81–1.71 (m, 1H, CH_2_), 1.63 (ddd, *J *= 13.8, 10.0, 3.6 Hz, 1H, CH_2_), 1.04 (s, 9H, 3 CH_3_). ^13^C NMR (75 MHz, Acetone‐d6) *δ* 179.85 (CO), 172.30 (CO), 169.31 (CO), 147.85 (CC), 142.10 (CC), 134.24 (Ar‐C), 131.12 (Ar‐C), 130.28 (Ar‐C), 128.34 (Ar‐C), 79.99 (CO), 60.54 (CH), 58.68 (CH), 58.60 (CH), 56.34 (CH_3_), 54.00 (CH), 48.67 (CH_2_), 40.58 (quat. C), 40.45 (CH_2_), 38.63 (CH_2_), 36.74 (CH_2_), 36.30 (CH_2_), 35.72 (CH_2_), 26.70 (3 CH_3_). HRMS (ESI) *m/z*: [M + Na]^+^ calculated for C_28_H_37_F_3_N_4_O_7_S: 653.2227; found: 653.2243.

**(2S,4R)−1‐{(S)−3,3‐Dimethyl‐2‐(2,2,2‐Trifluoroacetamido)Butanoyl}−4‐Methoxy‐N‐[(S)−1‐[((4‐Methoxybenzoyl)Oxy)Imino]−3‐[(S)−2‐Oxopyrrolidin‐3‐yl]Propan‐2‐yl]Pyrrolidine‐2‐Carboxamide (33).**

Obtained as a white solid (105 mg, 42% yield over five steps). ^1^H NMR (300 MHz, acetone) δ 8.18 (d, *J* = 9.2 Hz, 1H, NH), 8.07–7.93 (m, 5H, Ar‐H, NH), 7.07 (d, *J* = 2.1 Hz, 1H, Ar‐H), 7.04 (d, *J* = 2.3 Hz, 1H, Ar‐H), 6.84 (s, 1H, CH), 4.92 (ddt, *J* = 12.3, 8.5, 4.3 Hz, 1H, CH), 4.78–4.70 (m, 1H, CH), 4.52–4.40 (m, 1H, CH), 4.13 (dd, *J* = 4.6, 2.4 Hz, 1H, CH), 4.00 (d, *J* = 11.3 Hz, 1H, CH_2_), 3.90 (d, *J* = 1.0 Hz, 3H, CH_3_), 3.79 (dd, *J* = 11.2, 3.9 Hz, 1H, CH_2_), 3.29 (d, *J* = 2.2 Hz, 3H, CH_3_), 3.27 (s, 2H, CH_2_), 2.72 (m, 1H, CH_2_), 2.50–2.39 (m, 1H, CH_2_), 2.39–2.30 (m, 1H, CH_2_), 2.24 (ddd, *J* = 14.0, 11.9, 4.2 Hz, 1H, CH_2_), 1.85 (dd, *J* = 8.4, 3.5 Hz, 1H, CH_2_), 1.82–1.76 (m, 1H, CH_2_), 1.76–1.67 (m, 1H, CH_2_), 1.09 (s, 9H, 3 CH_3_), 1.02 (d, *J* = 20.2 Hz, 2H, CH_2_). ^13^C NMR (75 MHz, acetone) *δ* 179.73 (CO), 172.42 (CO), 169.22 (CO), 164.83 (CO), 163.56 (CN), 160.34 (Ar‐C), 132.36 (Ar‐C), 121.72 (Ar‐C), 114.89 (Ar‐C), 80.04 (CO), 60.38 (CH), 58.73 (CH), 56.38 (CH_3_), 55.99 (CH_3_), 54.01 (CH), 47.91 (CH_2_), 40.59 (quat. C), 38.23 (CH_2_), 36.81 (CH_2_), 35.65 (CH_2_), 34.83 (CH_2_), 26.72 (3 CH_3_). HRMS (ESI) *m/z*: [M + Na]^+^ calculated for C_29_H_38_F_3_N_5_O_8_: 664.2565; found: 664.2572.

**(2S,4S)‐N‐[(S)−1‐Cyano‐2‐[(S)−2‐Oxopyrrolidin‐3‐yl]Ethyl]−1‐{(S)−3,3‐Dimethyl‐2‐(2,2,2‐Trifluoroacetamido)Butanoyl}−4‐Phenylpyrrolidine‐2‐Carboxamide (34).**

Obtained as a white solid (166 mg, 48% over five steps). ^1^H NMR (500 MHz, cdcl3) *δ* 8.33 (d, *J *= 6.8 Hz, 1H, NH), 7.37–7.30 (m, 2H, Ar‐H), 7.24 (m, 3H, Ar‐H), 7.05 (dd, *J *= 21.7, 9.3 Hz, 1H, NH), 5.84 (d, 1H, NH), 4.88 (ddd, *J *= 10.4, 6.8, 5.8 Hz, 1H, CH), 4.72–4.63 (m, 1H, CH), 4.62 (m, 1H, CH), 4.33–4.20 (m, 1H, CH), 4.18 (m, 1H, CH), 3.87–3.76 (m, 1H, CH_2_), 3.73 (m, 1H, CH), 3.39–3.31 (m, 2H, CH_2_), 2.60–2.52 (m, 1H, CH_2_), 2.52–2.46 (m, 1H, CH_2_), 2.46–2.39 (m, 1H, CH_2_), 2.33 (m, 1H, CH_2_), 2.30–2.22 (m, 1H, CH_2_), 2.05–1.94 (m, 1H, CH_2_), 1.87 (m, 1H, CH_2_), 1.06 (s, 9H, 3 CH_3_). ^13^C NMR (126 MHz, cdcl3) δ 179.08 (CO), 171.42 (CO), 169.13 (CO), 139.04 (Ar‐C), 128.89 (Ar‐C), 128.74 (Ar‐C), 127.45 (Ar‐C), 127.05 (Ar‐C), 118.24 (CN), 60.10 (CH), 57.31 (CH), 54.31 (CH), 43.08 (CH_2_), 40.47 (quat. C), 39.53 (CH_2_), 37.82 (CH_2_), 36.24 (CH_2_), 35.61 (CH_2_), 33.94 (CH_2_), 29.67 (CH_2_), 28.48 (CH_2_), 26.29 (3 CH_3_).

### 4.2 Biochemical Assays

#### 4.2.1 Kinetic Assays

The assays were performed in a CLARIOstar microplate reader (BMG Labtech) and a Biotek Synergy HT microplate reader in a 96‐well white/black plate (Corning). The assay followed the release of 7‐amino‐4‐carbamoylmethylcoumarin (ACC) or 7‐amino‐4‐methylcoumarin (AMC) fluorophore from the substrates Ac‐Abu‐Tle‐Leu‐Gln‐ACC (QS1) [34] and Z‐Phe‐Arg‐AMC. The excitation and emission wavelengths for the substrate are 355 nm and 460 nm, respectively. The substrate hydrolysis rates (RFU/s) were determined.

#### 4.2.2 Jump Dilution Assay for the SARS‐CoV‐2 M^pro^

M^pro^ and inhibitors were incubated for 30 min at 100 nM and 200 nM in assay buffer. Subsequently, a 10‐fold dilution was performed, leading to a final concentration of [M^pro^] = 10 nM and [I] = 20 nM. The reaction started by adding the mixture to the QS1 substrate with a final concentration of 20 μM. The reaction was followed for approximately 30 min.

#### 4.2.3 Determination of the Inhibition Constants (*K*_i_) for the SARS‐CoV‐2 M^pro^

**Figure 8:**
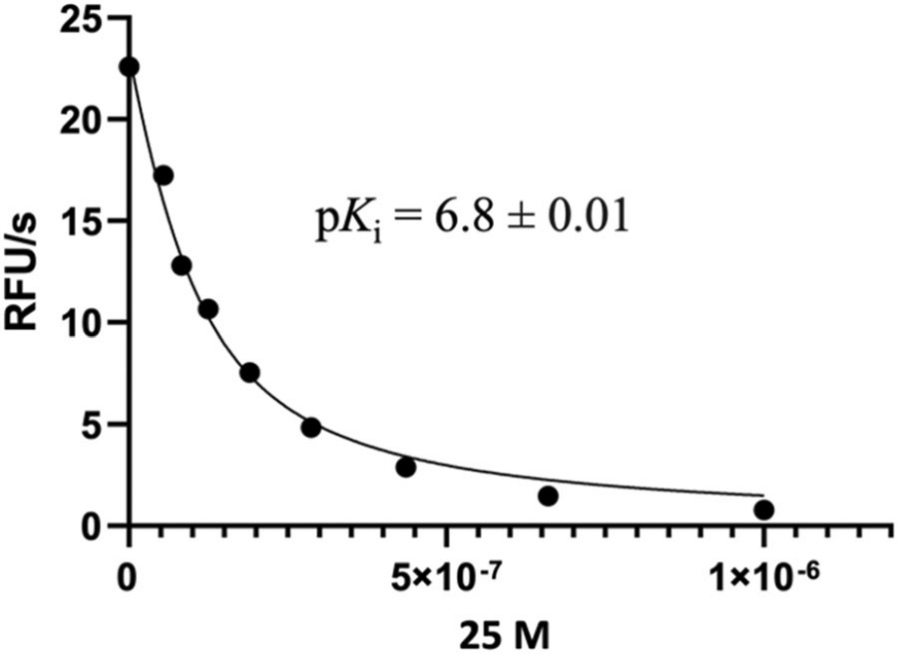
Assay curve for the determination of the inhibition constant (*K*_i_) for compound **22** against the WT M^pro^.

Inhibitors were prepared in assay buffer (20 mM PIPES, 100 mM NaCl, 1 mM EDTA, 0.7 M sodium citrate, 4 mM DTT, pH 7.2), with starting concentrations varying from 10 to 0.1 µM. The inhibitors were preincubated with M^pro^ (25 nM) for 30 min at 37°C. The reaction was started with the addition of the QS1 substrate with a final concentration of 20 µM. The inhibition constants were determined using the Morrison equation in the GraphPad Prism 8 software. An example of an inhibition curve is shown in Figure 8.

#### 4.2.4 Determination of *K*_inact_/*K*_I_ for Time‐Dependent Inhibitors for the SARS‐CoV‐2 M^pro^

**Figure 9:**
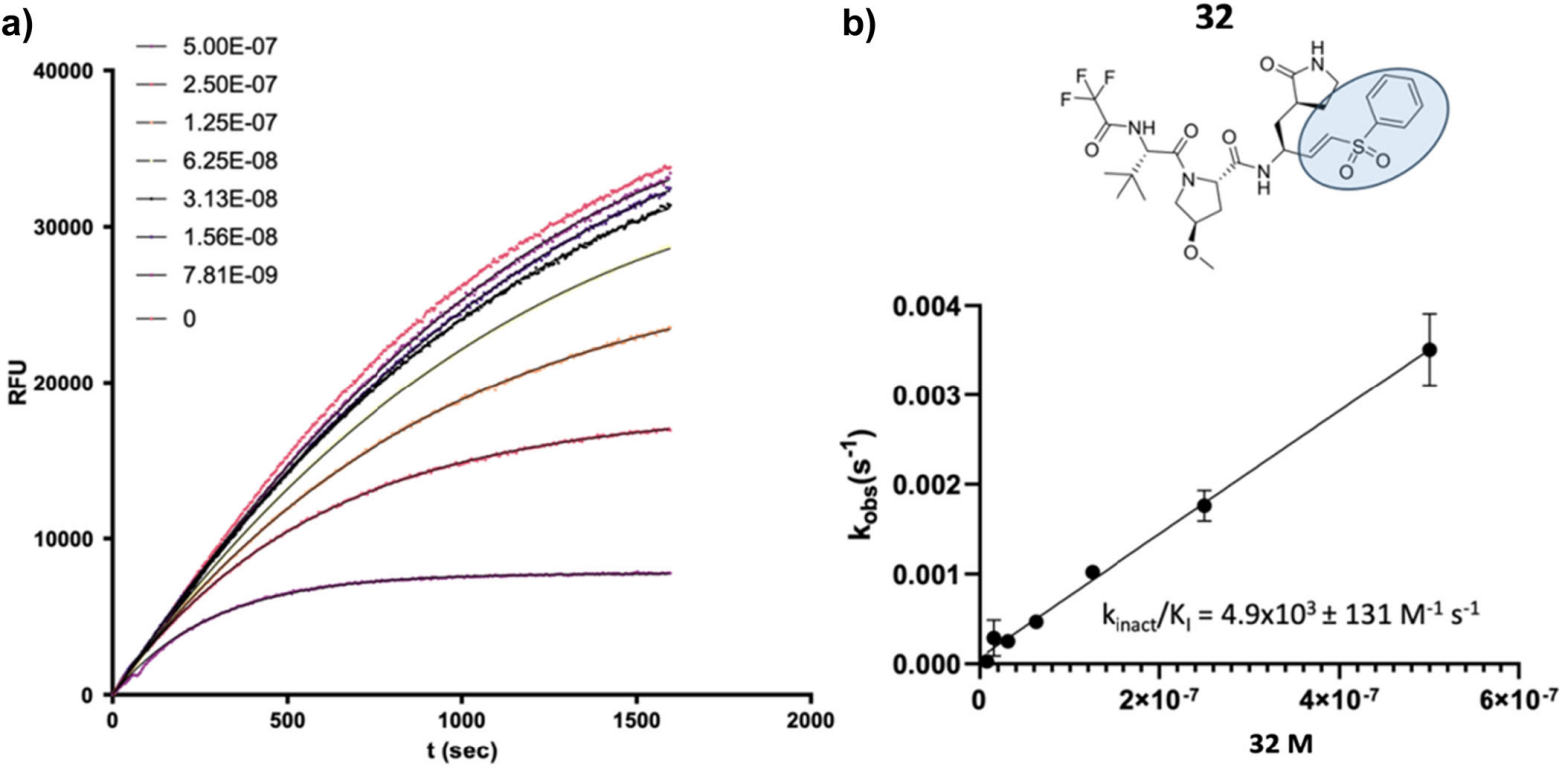
(a) Progress curve for the inhibition of WT M^pro^ by compound **32**. *k*_obs_ was determined by using Equation 1 for each inhibitor concentration. (b) The inhibitor's potency was obtained by linear regression of *k*_obs_ as a function of inhibitor concentration.

M^pro^ (25 nM) was added into wells containing a mixture of different concentrations of irreversible inhibitors, starting at 10 µM, and substrate QS1 (20 µM). The reaction was followed for 30 min. The progress curves were analyzed using GraphPad Prism 8 software. An example of irreversible inhibition for compound **32** is shown in Figure 9, where Equation (1) was utilized to obtain *k*_obs_ for each inhibitor concentration.

Equation (1) is used in GraphPad Prism for the determination of *k*_obs_ for irreversible inhibitors. *P* is the product formation, *v*_o_ is the initial reaction rate, *v*_s_ is the steady‐state velocity, and t is the time.

(1) $$[P]=v_{s}\;.t+\frac{{(v}_{0}-v_{s})}{k_{\mathrm{obs}}}\;.(1-e^{{-k}_{\mathrm{obs}}.t}).$$

#### 4.2.5 Determination of the Inhibition Constants (*K*_I_) for the Human Cathepsins

Inhibitors were prepared in assay buffer, which vary depending on the enzyme (Table 2), with starting concentrations varying from 10 to 0.1 µM. The inhibitors were preincubated with the cathepsins for 5 min at room temperature. The reaction started with the addition of the Z‐FR‐AMC substrate solution, with a concentration equal to the enzyme's *K*_M_. The inhibition constants were determined using the Morrison equation in the GraphPad Prism 8 software. Notably, none of the tested compounds exhibited fluorescence at the wavelengths employed in the assay.

**Table 2:** Parameters used in the kinetics assays for different cathepsins.

Enzyme	[Enzyme] nM	Substrate	Assay buffer	Enzyme activation time	Enzyme activation temperature
*h*CatL	1.9	Z‐FR‐AMC	100 mM sodium acetate pH 5.5	20 min	0°C
*h*CatS	2.0	Z‐FR‐AMC	100 mM sodium citrate pH 6.0	1 h	0°C
*h*CatB	1.0	Z‐FR‐AMC	100 mM sodium phosphate pH 6.0	30 min	0°C

### 4.3 Molecular Modeling

Since the inhibitors explored in this study were designed to form a covalent adduct with the catalytic cysteine (Cys145) of M^pro^, we focused exclusively on covalent docking. Previous studies suggest that this represents the most critical binding state for reversible covalent inhibitors [35, 36].

Covalent docking was performed using GOLD [37]. The M^pro^ structures were obtained from the protein data bank (PDB codes: 7RFS and 7LB7). The protonation states of the amino acids were assigned using PDB2PQR [38] at the same pH as the biochemical assays. For the ligands, p*K*_a_ values were predicted using MolGpKa [39]. The initial 3D conformations of the compounds were generated from their SMILES representations using OMEGA [40].

All ligands were modeled in their covalently bound form, with an open valence electron (S^−^) on both the ligand and the catalytic Cys145 of M^pro^, serving as the linking atoms. The binding pocket was defined as a sphere with a 10 Å radius centered on the sulfur atom of Cys145. For each compound, 20 docking poses were generated and visually inspected. The evaluation focused on key molecular interactions, particularly the hydrogen bonds with the backbone of His164 and Glu166 and the side chains of His163 and Glu166, as well as π‐stacking interactions with His41 when applicable and S2 pocket occupation.

The MD simulations were initiated from the covalent docking poses. Ligand partial charges were assigned using the AM1‐BCC method, and the ligands were parameterized with GAFF2. The protein was treated with the ff14SB [41] force field, and the systems were solvated in a truncated octahedral TIP3P [42] water box with Na^+^ counterions for neutralization. All simulations were performed using the Amber20 [43] suite of programs. System minimization was carried out for 10,000 cycles using the steepest descent and conjugate gradient algorithms. Subsequently, the systems were gradually heated to 300 K in multiple steps, controlled by a Langevin thermostat. Finally, 100 ns of MD simulations were conducted under NPT ensemble conditions.

### 4.4 Metabolic Stability Against Liver Microsomes

Metabolic stability measurement was performed as described before [44, 45]. Pooled liver microsomes from female Sprague–Dawley were purchased from Sigma‐Aldrich (Germany). To determine the metabolic stability, liver microsomal proteins (0.2 mg/mL) were supplemented with NADPH (5 mM) in Dulbecco's Phosphate Buffered Saline (Sigma‐Aldrich, Germany) and preincubated at 37°C for 15 min. Test compounds (100 µM) were added and incubated at 37°C. Aliquots were removed at various time points. The reaction was terminated by the addition of acetonitrile, and the samples were cooled with ice for 15 min before centrifugation (3000*g* at 4°C for 20 min). The supernatants were used for further analysis. The loss of parent compound was monitored by HPLC on a Jasco HPLC system with UV detector and RP‐18 column (ReproSil‐Pur‐ODS, Dr. Maisch GmbH, Germany, 3 μm, 50 mm × 2 mm) using the following method: eluent A, water (+0.1% TFA); eluent B. acetonitrile (+0.1% TFA); flow rate, 1 mL/min; gradient, 1% B (0.2 min), 100% B (3.5 min), 100% B (4.5 min), 1% B (4.6 min), 1% B (5 min). The metabolic stability was determined by dividing the peak areas of the unaltered parent compound in the metabolized sample by the peak areas of the parent compound in the reference sample. The activity of the microsomal preparations was verified by using a positive control (testosterone).

## Conflicts of Interest

The authors declare no conflicts of interest.

## Supporting information

ArchPharm_SupplMat_InChI_2020.

SIFinalVersion.
