# Supplementary material for: Exploring Nirmatrelvir Derivatives Through P2 Substituent Modifications and Warhead Innovations Targeting the Main Protease of SARS‐CoV‐2
Source: Arch Pharm (Weinheim). 2025 Nov 29;358(11):e70158. doi: 10.1002/ardp.70158 (PMC12664123; doi:10.1002/ardp.70158)
Supplement: Supplementary file 1 — ArchPharm_SupplMat_InChI_2020. [file ARDP-358-e70158-s002.doc]

**Supplemental Material: Novel Compounds and Biological Screening Results**

**Exploring Nirmatrelvir Derivatives through P2 Substituent Modifications and Warhead Innovations to Enhance SARS-CoV-2 M^pro^ Targeting**

Felipe Cardoso Prado Martins^1,2^, Johannes Lang^2^, Fernanda dos Reis Rocho^1^, Xianxian Wang^2^, Vinícius Bonatto^1^, Jerônimo Lameira^1^, Christian Klein^2^, Carlos Alberto Montanari^1^,*

^1^Medicinal and Biological Chemistry Group, São Carlos Institute of Chemistry, University of São Paulo, Avenue Trabalhador Sancarlense 400, 13566-590, São Carlos, São Paulo, Brazil.

^2^Medicinal Chemistry, Institute of Pharmacy and Molecular Biotechnology IPMB, Heidelberg University, Im Neuenheimer Feld 364, 69120, Heidelberg, Germany.

* Corresponding Author.

Carlos Alberto Montanari, Medicinal and Biological Chemistry Group, São Carlos Institute of Chemistry, University of São Paulo, Avenue Trabalhador Sancarlense 400, 13566-590, São Carlos, São Paulo, Brazil.

E-mail: carlos.montanari@usp.br; Phone: +55-16-3373-8060.

| **Compound No.** | **InChI** | **Biological Activity (p*K*_i_)^a^** |
| --- | --- | --- |
| 22 | InChI=1S/C21H32F3N5O6/c1-20(2,3)15(28-19(33)21(22,23)24)18(32)29-10-13(35-4)8-14(29)17(31)27-12(9-26-34)7-11-5-6-25-16(11)30/h9,11-15,34H,5-8,10H2,1-4H3,(H,25,30)(H,27,31)(H,28,33)/b26-9+/t11-,12-,13+,14-,15+/m0/s1 | 6.8 ± 0.01 |
| 23 | InChI=1S/C26H34F3N5O5/c1-25(2,3)20(33-24(38)26(27,28)29)23(37)34-14-17(15-7-5-4-6-8-15)12-19(34)22(36)32-18(13-31-39)11-16-9-10-30-21(16)35/h4-8,13,16-20,39H,9-12,14H2,1-3H3,(H,30,35)(H,32,36)(H,33,38)/b31-13+/t16-,17+,18-,19-,20+/m0/s1 | 7.3 ± 0.01 |
| 24 | InChI=1S/C23H34F3N5O6/c1-5-8-37-15-10-16(19(33)29-14(11-28-36)9-13-6-7-27-18(13)32)31(12-15)20(34)17(22(2,3)4)30-21(35)23(24,25)26/h5,11,13-17,36H,1,6-10,12H2,2-4H3,(H,27,32)(H,29,33)(H,30,35)/b28-11+/t13-,14-,15+,16-,17+/m0/s1 | 7.3 ± 0.08 |
| 25 | InChI=1S/C23H32F3N5O6/c1-5-8-37-15-10-16(19(33)29-14(11-28-36)9-13-6-7-27-18(13)32)31(12-15)20(34)17(22(2,3)4)30-21(35)23(24,25)26/h1,11,13-17,36H,6-10,12H2,2-4H3,(H,27,32)(H,29,33)(H,30,35)/b28-11+/t13-,14-,15+,16-,17+/m0/s1 | 7.5 ± 0.04 |
| 26 | InChI=1S/C22H34F3N5O6/c1-5-36-14-9-15(18(32)28-13(10-27-35)8-12-6-7-26-17(12)31)30(11-14)19(33)16(21(2,3)4)29-20(34)22(23,24)25/h10,12-16,35H,5-9,11H2,1-4H3,(H,26,31)(H,28,32)(H,29,34)/b27-10+/t12-,13-,14+,15-,16+/m0/s1 | 7.0 ± 0.02 |
| 27 | InChI=1S/C26H40F3N5O5/c1-25(2,3)20(33-24(38)26(27,28)29)23(37)34-14-17(15-7-5-4-6-8-15)12-19(34)22(36)32-18(13-31-39)11-16-9-10-30-21(16)35/h13,15-20,39H,4-12,14H2,1-3H3,(H,30,35)(H,32,36)(H,33,38)/b31-13+/t16-,17+,18-,19-,20+/m0/s1 | 7.0 ± 0.02 |
| 28 | InChI=1S/C22H33F3N6O6/c1-11(32)28-14-8-15(18(34)29-13(9-27-37)7-12-5-6-26-17(12)33)31(10-14)19(35)16(21(2,3)4)30-20(36)22(23,24)25/h9,12-16,37H,5-8,10H2,1-4H3,(H,26,33)(H,28,32)(H,29,34)(H,30,36)/b27-9+/t12-,13-,14+,15-,16+/m0/s1 | 7.0 ± 0.03 |
| 29 | InChI=1S/C27H36F3N5O6/c1-26(2,3)21(34-25(39)27(28,29)30)24(38)35-14-19(41-15-16-7-5-4-6-8-16)12-20(35)23(37)33-18(13-32-40)11-17-9-10-31-22(17)36/h4-8,13,17-21,40H,9-12,14-15H2,1-3H3,(H,31,36)(H,33,37)(H,34,39)/b32-13+/t17-,18-,19+,20-,21+/m0/s1 | 6.7 ± 0.02 |
| 30 | InChI=1S/C28H36F4N4O7S/c1-27(2,3)22(35-26(40)28(30,31)32)25(39)36-15-18(43-4)14-20(36)24(38)34-17(12-16-10-11-33-23(16)37)13-21(29)44(41,42)19-8-6-5-7-9-19/h5-9,13,16-18,20,22H,10-12,14-15H2,1-4H3,(H,33,37)(H,34,38)(H,35,40)/b21-13+/t16-,17-,18+,20-,22+/m0/s1 | 8.1 ± 0.01 |
| 31 | InChI=1S/C21H30F3N5O5/c1-20(2,3)15(28-19(33)21(22,23)24)18(32)29-10-13(34-4)8-14(29)17(31)27-12(9-25)7-11-5-6-26-16(11)30/h11-15H,5-8,10H2,1-4H3,(H,26,30)(H,27,31)(H,28,33)/t11-,12-,13+,14-,15+/m0/s1 | 8.8 ± 0.01 |
| 32^b^ | InChI=1S/C28H37F3N4O7S/c1-27(2,3)22(34-26(39)28(29,30)31)25(38)35-16-19(42-4)15-21(35)24(37)33-18(14-17-10-12-32-23(17)36)11-13-43(40,41)20-8-6-5-7-9-20/h5-9,11,13,17-19,21-22H,10,12,14-16H2,1-4H3,(H,32,36)(H,33,37)(H,34,39)/b13-11+/t17-,18+,19+,21-,22+/m0/s1 | *K*_inact_/K_I_ = 4.4x10^3^ M^-1^ s-^1^ (± 500 M^-1^ s-^1^) |
| 33^b^ | InChI=1S/C29H38F3N5O8/c1-28(2,3)22(36-27(42)29(30,31)32)25(40)37-15-20(44-5)13-21(37)24(39)35-18(12-17-10-11-33-23(17)38)14-34-45-26(41)16-6-8-19(43-4)9-7-16/h6-9,14,17-18,20-22H,10-13,15H2,1-5H3,(H,33,38)(H,35,39)(H,36,42)/b34-14+/t17-,18-,20+,21-,22+/m0/s1 | *K*_inact_/K_I_ = 7.7x10^4^ M^-1^ s^-1^ (± 1930 M^-1^ s-^1^) |
| 34 | InChI=1S/C26H32F3N5O4/c1-25(2,3)20(33-24(38)26(27,28)29)23(37)34-14-17(15-7-5-4-6-8-15)12-19(34)22(36)32-18(13-30)11-16-9-10-31-21(16)35/h4-8,16-20H,9-12,14H2,1-3H3,(H,31,35)(H,32,36)(H,33,38)/t16-,17+,18-,19-,20+/m0/s1 | 8.9 ± 0.01 |

^a^ Inhibitors were prepared in assay buffer (20 mM PIPES, 100 mM NaCl, 1 mM EDTA, 0.7 M sodium citrate, 4 mM DTT, pH 7.2), with starting concentrations varying from 10 to 0.1 µM. The inhibitors were preincubated with M^pro^ (25 nM) for 30 min at 37ºC. The reaction was started with the addition of the QS1 substrate with a final concentration of 20 µM. The inhibition constants were determined using the Morrison equation in the GraphPad Prism 8 software.

^b^ M^pro^ (25 nM) was added into wells containing a mixture of different concentrations of irreversible inhibitors, starting at 10 µM, and substrate QS1 (20 µM). The reaction was followed for 30 min. The progress curves were analyzed using GraphPad Prism 8 software.
