# Supplementary material for: Exploring Nirmatrelvir Derivatives Through P2 Substituent Modifications and Warhead Innovations Targeting the Main Protease of SARS‐CoV‐2
Source: Arch Pharm (Weinheim). 2025 Nov 29;358(11):e70158. doi: 10.1002/ardp.70158 (PMC12664123; doi:10.1002/ardp.70158)
Supplement: Supplementary file 2 — SIFinalVersion. [file ARDP-358-e70158-s001.docx]

Supplementary Information

**Exploring Nirmatrelvir Derivatives through P2 Substituent Modifications and Warhead Innovations to Enhance SARS-CoV-2 M^pro^ Targeting**

Felipe Cardoso Prado Martins^1,2^, Johannes Lang^2^, Fernanda dos Reis Rocho^1^, Xianxian Wang^2^, Vinícius Bonatto^1^, Jerônimo Lameira^1^, Christian Klein^2^, Carlos Alberto Montanari^1,^*

^1^Medicinal and Biological Chemistry Group, São Carlos Institute of Chemistry, University of São Paulo, Avenue Trabalhador Sancarlense 400, 13566-590, São Carlos, São Paulo, Brazil.

^2^Medicinal Chemistry, Institute of Pharmacy and Molecular Biotechnology IPMB, Heidelberg University, Im Neuenheimer Feld 364, 69120, Heidelberg, Germany.

* Corresponding Author.

Carlos Alberto Montanari, Medicinal and Biological Chemistry Group, São Carlos Institute of Chemistry, University of São Paulo, Avenue Trabalhador Sancarlense 400, 13566-590, São Carlos, São Paulo, Brazil.

E-mail: carlos.montanari@usp.br; Phone: +55-16-3373-8060.

**Table of Contents**

Abbreviations

Table of final compounds

Full characterization of final compounds

**Abbreviations.**

MeCN, acetonitrile; DMF, *N*,*N*-dimethylformamide; DCM, methylene chloride; Boc, *tert*-butoxycarbonyl; DIPEA, *N*,*N*-diisopropylethylamine; DMSO, dimethylsulfoxide; HATU, 1- [bis(dimethylamino)methylene]-1*H*-1,2,3-triazolo[4,5-*b*]pyridinium 3-oxide hexafluorophosphate; KHMDS, potassium bis(trimethylsilyl)amide; LHMDS, lithium bis(trimethylsilyl)amide; TFA, trifluoroacetic acid; THF, tetrahydrofuran;

**Table of final compounds.**

| Manuscript code | NeqCode | SMILES |
| --- | --- | --- |
| 22 | Neq1220 | CO[C@@H]1C[C@@H](C(N[C@@H](C[C@@H]2CCNC2=O)/C=N/O)=O)N(C([C@H](C(C)(C)C)NC(C(F)(F)F)=O)=O)C1 |
| 23 | Neq1223 | O=C([C@H](C(C)(C)C)NC(C(F)(F)F)=O)N(C1)[C@H](C(N[C@@H](C[C@@H]2CCNC2=O)/C=N/O)=O)C[C@H]1C3=CC=CC=C3 |
| 24 | Neq1224 | O=C([C@H](C(C)(C)C)NC(C(F)(F)F)=O)N(C1)[C@H](C(N[C@@H](C[C@@H]2CCNC2=O)/C=N/O)=O)C[C@H]1OCC=C |
| 25 | Neq1225 | O=C([C@H](C(C)(C)C)NC(C(F)(F)F)=O)N(C1)[C@H](C(N[C@@H](C[C@@H]2CCNC2=O)/C=N/O)=O)C[C@H]1OCC#C |
| 26 | Neq1226 | O=C([C@H](C(C)(C)C)NC(C(F)(F)F)=O)N(C1)[C@H](C(N[C@@H](C[C@@H]2CCNC2=O)/C=N/O)=O)C[C@H]1OCC |
| 27 | Neq1228 | O=C([C@H](C(C)(C)C)NC(C(F)(F)F)=O)N(C1)[C@H](C(N[C@@H](C[C@@H]2CCNC2=O)/C=N/O)=O)C[C@H]1C3CCCCC3 |
| 28 | Neq1234 | O=C([C@H](C(C)(C)C)NC(C(F)(F)F)=O)N(C1)[C@H](C(N[C@@H](C[C@@H]2CCNC2=O)/C=N/O)=O)C[C@H]1NC(C)=O |
| 29 | Neq1235 | O=C([C@H](C(C)(C)C)NC(C(F)(F)F)=O)N(C1)[C@H](C(N[C@@H](C[C@@H]2CCNC2=O)/C=N/O)=O)C[C@H]1OCC3=CC=CC=C3 |
| 30 | Neq1229 | CO[C@@H]1C[C@@H](C(N[C@@H](C[C@@H]2CCNC2=O)/C=C(F)/S(=O)(C3=CC=CC=C3)=O)=O)N(C([C@H](C(C)(C)C)NC(C(F)(F)F)=O)=O)C1 |
| 31 | Neq1233 | CO[C@@H]1C[C@@H](C(N[C@@H](C[C@@H]2CCNC2=O)C#N)=O)N(C([C@H](C(C)(C)C)NC(C(F)(F)F)=O)=O)C1 |
| 32 | Neq1230 | CO[C@@H]1C[C@@H](C(N[C@@H](C[C@@H]2CCNC2=O)/C=C/S(=O)(C3=CC=CC=C3)=O)=O)N(C([C@H](C(C)(C)C)NC(C(F)(F)F)=O)=O)C1 |
| 33 | Neq1236 | O=C([C@H](C(C)(C)C)NC(C(F)(F)F)=O)N(C1)[C@H](C(N[C@@H](C[C@@H]2CCNC2=O)/C=N/OC(C3=CC=C(OC)C=C3)=O)=O)C[C@H]1OC |
| 34 | Neq1248 | O=C([C@H](C(C)(C)C)NC(C(F)(F)F)=O)N(C1)[C@H](C(N[C@@H](C[C@@H]2CCNC2=O)C#N)=O)C[C@H]1C3=CC=CC=C3 |

__

__

__

__

__

__

__

__

__

__

__

__

__

__

__

__

__

__

__

__

__

__

__

__

__
